# Mechanism of Cardiac Arrest in Fatal Anaphylaxis

**DOI:** 10.1111/cea.70289

**Published:** 2026-03-24

**Authors:** Ben A. McKenzie, Stuart D. Marshall, Lena A. Sanci, Catriona Moynihan, Chris Selman, Jo A. Douglass

**Affiliations:** ^1^ Department of Medicine, Melbourne Medical School University of Melbourne Melbourne Victoria Australia; ^2^ National Allergy Centre of Excellence (NACE), Parkville Victoria Australia; ^3^ Department of Critical Care, Melbourne Medical School University of Melbourne Victoria Australia; ^4^ Department of Emergency Medicine, Royal Melbourne Hospital Parkville Victoria Australia; ^5^ Department of General Practice and Primary Care, Melbourne Medical School University of Melbourne Victoria Australia; ^6^ Department of Allergy and Immunology, Royal Melbourne Hospital Parkville Victoria Australia

**Keywords:** anaphylaxis, cardiac arrest, death, drug allergy, food allergy, insect allergy

## Abstract

**Introduction:**

Anaphylaxis is a short duration, potentially catastrophic allergic reaction where a rapid return to a person's baseline can be expected if vital organ function can be supported. Fatal anaphylaxis is a rare but potentially preventable cause of death at all ages. The three main mechanisms of rapid organ system failure leading to cardiac arrest in anaphylaxis are upper airway obstruction, lower respiratory obstruction principally from bronchospasm and cardiovascular failure. We aimed to measure the frequency and timing of each organ failure type leading to fatal anaphylaxis to further inform treatment recommendations.

**Methods:**

We performed a population‐based retrospective cohort analysis for the period 1 January 2003 to 31 December 2022 using Australian clinical data contained within the National Coronial Information System. The primary outcome was the primary organ that failed leading to initial physiological decompensation. Secondary objectives were to compare the time course of deterioration and complications between allergen groups. Multivariate logistic regression was used to quantify adjusted odds ratios between allergen categories for each of the three organ failure types.

**Results:**

There were 371 anaphylaxis deaths for the study period (mean age 55.7 years, male 58.5%) with the primary outcome recorded in 250 (67.4%). In overall all‐cause anaphylaxis fatalities, the most common organ failure was bronchospasm‐induced respiratory failure (*n* = 153, 61.2%). Cardiovascular failure was less common (*n* = 73, 29.2%) and upper airway obstruction occurred in 24 (9.6%). There were significant differences in the primary organ system failure according to allergen trigger categories. Fatal food anaphylaxis was exclusively associated with respiratory failure (bronchospasm 94.8%, upper airway 5.2%).

**Conclusion:**

We found that respiratory failure rather than cardiovascular failure was the most common cause of cardiorespiratory arrest in fatal anaphylaxis. Death from food‐triggered anaphylaxis appears to be exclusively a primary respiratory event. International anaphylaxis guidelines require appropriate emphasis on respiratory symptoms and treatment to further reduce the risk of anaphylaxis fatalities.

## Key Messages

- Respiratory failure, mainly from bronchospasm, is the most frequent cause of cardiac arrest in fatal anaphylaxis.
- Food‐triggered anaphylaxis deaths are likely to be exclusively from respiratory failure, principally from bronchospasm.
- The type of organ failure leading to cardiorespiratory arrest differs significantly between allergen triggers.

## Graphical abstract

This population‐based retrospective cohort study of clinical deterioration in anaphylaxis deaths identifies bronchospasm as the preponderant cause of cardiac arrest, especially in food and drug allergen exposures. The clinical implication is that anaphylaxis guidelines require appropriate emphasis on respiratory involvement and treatment to prevent deaths.

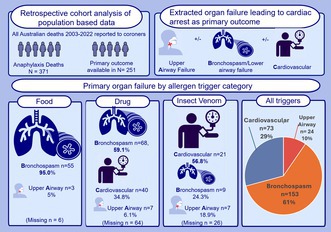

## 1 Introduction

Anaphylaxis is a potentially catastrophic, sudden onset multiorgan allergic reaction that is of short duration, usually lasting less than 4 h [1]. It occurs when a sensitised person is exposed to an allergen, which is most commonly a drug, food or insect venom [2]. During anaphylaxis, release of short‐acting inflammatory mediators such as leukotrienes, platelet activating factor, prostaglandin D2 and histamine occur with profound cardiovascular and respiratory effects [3]. Anaphylaxis has a spectrum of severity [4, 5] but a rapid return to a person's baseline can be expected, even in severe cases, if vital organ function can be supported for the brief duration of the disease process.

Even though rare, anaphylaxis is an important cause of unexpected and potentially preventable death at all ages [6]. The three main mechanisms of rapid organ failure in anaphylaxis are upper airway obstruction, lower respiratory obstruction due to bronchospasm leading to ventilatory failure and cardiovascular failure observed as hypotension [7]. Understanding the frequency and timing of each organ system failure in fatal anaphylaxis is likely to inform treatment recommendations by identifying high‐risk symptoms, thereby enabling the appropriate direction of time critical organ‐specific support.

Whole population fatal anaphylaxis data have been studied previously in various jurisdictions, but none have deliberately examined the type of organ failure primarily responsible for cardiorespiratory arrest in anaphylaxis fatalities [7, 8, 9, 10]. To address this knowledge gap, our primary objective was to examine differences in primary organ failure that develop in patients who have fatal anaphylaxis after being exposed to different allergen trigger categories. A secondary objective was to compare the time course of symptom onset and complications according to allergen exposure.

## 2 Methods

### 2.1 Study Setting and Design

We performed a population‐based retrospective cohort analysis of consecutive anaphylaxis deaths reported to all Australian coroners for the period 1 January 2003 to 31 December 2022 (20 years). In Australia, there is mandatory reporting of unexpected deaths to a coroner whose responsibility is to determine the identity, cause and circumstances surrounding each reported death. This study was performed in accordance with Strengthening the Reporting of Observational Studies in Epidemiology (STROBE) reporting guidelines [11] and was approved by the University of Melbourne Human Research Ethics Committee 2023‐27190‐43989‐3 and the Victorian Department of Justice Human Research Ethics Committee CF/23/26492. As a condition of ethics approval, death counts of less than five cannot be directly reported. Where this has occurred in tabular reporting, we have abbreviated this to Not Reportable (NR).

### 2.2 Data Source and Cohort Derivation

We retrieved Australian data from the National Coronial Information System (NCIS), a national database that contains clinical information regarding all deaths reported to Australian coroners. The NCIS contains clinical information collected during the coronial review process that is likely to include eyewitness accounts of events, summary of medical records, autopsy and toxicology reports. Cases were identified in the NCIS for screening using the following search terms: (a) cause of death field search using search term “anaphyl*” in all primary and antecedent causes of death listed; (b) ICD 10 search—T78 Anaphylaxis (includes food), T805 Serum Anaphylaxis, T886 Anaphylaxis to drug correctly administered, T634 Arthropod Anaphylaxis, Y575 Drug anaphylaxis (c) Keyword free text search function of reports, Anaphyl*.

Two emergency medicine specialists (CM and BM), trained in anaphylaxis identification, independently assessed deaths for inclusion as confirmed anaphylaxis. For cases where anaphylaxis was an uncertain cause of death for either extractor, a formal third person review by a specialist allergy and immunologist (JD) occurred.

### 2.3 Baseline Characteristics

Baseline characteristics were collected including age, sex, history of asthma/chronic obstructive pulmonary disease (COPD), any antihypertensive use, whether the patient was known to be allergic to the causative agent and American Society of Anaesthesiologists Physical Status Classification System (ASA) Score. The latter is a scoring system to measure a patient's level of systemic disease (comorbidities) prior to the physiological challenge of anaesthesia to help assess perioperative risk.

### 2.4 Outcome Measures

The primary outcome was primary organ failure causing anaphylaxis death, which was one of either upper airway obstruction, bronchospasm or cardiovascular failure. We identified a set of criteria to determine the presence of each organ failure using expert consensus by a panel of clinicians comprising Anaesthesia, Emergency Medicine, Primary Care, and Allergy and Immunology specialists. The panel used a three‐step process to develop descriptors for each organ involvement. The first was mapping descriptors from nine anaphylaxis‐grading systems and two organ‐specific (asthma and laryngotracheobronchitis) severity descriptors and categorising them into each organ failure type. The second step involved refining criteria to reduce error from potential overlap of symptoms/signs including consideration of published data referencing organ failure interrelation and symptom overlap. The third step was revision from individual domains of expertise from each panel specialty. The table of descriptors used is published in Table S1 with further detail on derivation in the study protocol (Supporting Information).

The final criteria were used for independent double data extraction by two critical care specialists (BM and CM) with adjudication from the expert panel, where required, to resolve differences. Once organ involvement was identified prior to death, data extractors were required to clinically determine the primary organ that failed leading to initial physiological decompensation with the option to include secondary organ failure if more than one organ failure was deemed to be present. If a case could not be categorised due to overlap of symptoms and insufficient clinical details, then this was coded as missing outcome data.

The secondary objectives were to compare the following outcomes by patient allergen exposure: complications and interventions, and time sequence from (a) allergen exposure to symptom onset; (b) symptom onset (any organ symptom signifying the onset of the allergic reaction) to severe symptoms (which correlate with hypoxia and hypotension, see notes Table 4); (c) severe symptoms to cardiorespiratory arrest; and (d) cardiorespiratory arrest to death. Other variables collected include timing of tracheal intubation; time to tracheal intubation from cardiorespiratory arrest, adrenaline administration prior to arrest, whether the patient experienced vomiting or aspiration, and the presence of complications including front of neck access (FONA, surgical airway), difficult airway (surgical airway or more than two intubation attempts), pneumothorax, cardioversion or myocardial infarction.

### 2.5 Exposure

Where possible the likely anaphylaxis trigger was identified as either drug, radiocontrast media, insect venom or food as the most common allergens in Australia. We also collected data on other and unknown allergen triggers.

### 2.6 Statistical Analysis

Data analysis was performed using Stata Statistical Software: Release 19 (StatCorp. 2025). Baseline categorical variables were summarised as number and percentage, and continuous variables as mean, standard deviation and range. For the primary outcome, we used the allergen trigger as the exposure of interest and primary organ failure recorded as a binary outcome variable, defined as whether the primary organ failure was respiratory or cardiovascular as the outcome in three models where each type of organ failure was a separate binary outcome. Prior to database lock, we elicited key confounders from clinical expertise (age) and effect modifiers (age, sex, asthma/COPD status, antihypertensive use and ASA score) to include in the analysis of the primary outcome since our interest was in estimating the effect of allergen exposure on primary organ failure. We used a logistic regression model adjusting for these confounders and effect modifiers (which included an interaction term between the exposure and effect modifier) to estimate an adjusted odds ratio (OR) reported with 95% confidence intervals (CIs) and *p* value. An adjusted marginal OR was estimated following model fitting using g‐computation. Given the exposure variable has multiple categories, we presented each pairwise OR.

Continuous secondary outcomes (namely timing of symptoms) were compared between exposure groups using a median difference estimated using quantile regression. Similarly, binary outcomes were compared between exposure groups using an OR using logistic regression. We did not adjust for confounders in these models, as the aim was simply to describe and compare outcomes between exposure groups.

We conducted a planned sensitivity analysis using multiple imputation using chained equations to handle missing data. Imputations were generated using chained equations with 50 imputed datasets and 10 iterations between each imputation. We included all variables in the analysis model and auxiliary variables in the imputation model, including all interaction terms to ensure the imputation model was compatible with the analysis model. Estimates of interest were obtained using Rubin's rules [12].

## 3 Results

Search of the NCIS revealed the following results: ICD search 471 deaths; cause of death search 400 deaths and keyword search 2028 deaths. Of the 2899 cases identified, reviewers agreed that 359 cases should be included, and 29 were referred for specialist adjudication of which 12 were included. The final cohort consisted of 371 anaphylaxis deaths (mean age [standard deviation, SD] 55.7 [20.4] years), of which 217 (59%) were male (Table 1).

**TABLE 1:** Baseline characteristics of anaphylaxis deaths by allergen exposure.

	Overall sample	Allergen exposure*			
		Drug	Insect venom	Food	Radiocontrast
Number of eligible patients	*N* = 371	*N* = 179	*N* = 63	*N* = 64	*N* = 45
Biological sex					
Male	217 (58.5%)	97 (54.2%)	55 (87.3%)	36 (56.2%)	22 (48.9%)
Female	154 (41.5%)	82 (45.8%)	8 (12.7%)	28 (43.8%)	23 (51.1%)
Age (years)	Mean = 55.7 (SD = 20.4)	Mean = 62.3 (SD = 15.8)	Mean = 54.1 (SD = 16.5)	Mean = 30.2 (SD = 17.4)	Mean = 68.7 (SD = 15.2)
Age group					
0–4 years	NR	NR	NR	NR	NR
5–17 years	19 (5.1%)	NR	NR	17 (26.6%)	NR
18–39 years	58 (15.7%)	16 (9.0%)	12 (19.0%)	27 (42.2%)	NR
40–59 years	115 (31.1%)	53 (29.8%)	27 (42.9%)	15 (23.4%)	13 (28.9%)
60+ years	178 (48.1%)	109 (61.2%)	24 (38.1%)	NR	32 (71.1%)
Any history of asthma/COPD					
Yes	136 (48.6%)	62 (44.0%)	8 (21.1%)	49 (90.7%)	7 (22.6%)
No	144 (51.4%)	79 (56.0%)	30 (78.9%)	5 (9.3%)	24 (77.4%)
Missing observations	91	38	25	10	14
History of antihypertensives					
No	291 (78.4%)	130 (72.6%)	48 (76.2%)	61 (95.3%)	34 (75.6%)
ASA Score					
1	35 (10.1%)	14 (8.4%)	16 (27.6%)	NR	NR
2	161 (46.5%)	56 (33.5%)	34 (58.6%)	49 (81.7%)	14 (31.8%)
3	116 (33.5%)	71 (42.5%)	8 (13.8%)	6 (10.0%)	23 (52.3%)
4	34 (9.8%)	26 (15.6%)	0 (0.0%)	NR	6 (13.6%)
Missing observations	25	12	5	4	1
Known allergen to patient					
Yes	110 (37.0%)	25 (17.1%)	28 (57.1%)	52 (85.2%)	NR
No	187 (63.0%)	121 (82.9%)	21 (42.9%)	9 (14.8%)	33 (94.3%)
Missing observations	74	33	14	3	10

* Missing allergen data/other allergen exposure (*n* = 20) data not included in analysis and not displayed except in overall sample. NR, not reportable (Counts less than five are not directly reported as a condition of the Victorian Department of Justice Human Ethics Approval).

The level of initial agreement between extractors was 64.7% (240 deaths) in assessing the primary outcome (upper airway, bronchospasm, cardiovascular or unable to be determined) prior to the consensus process. Of the remaining 131 deaths, consensus was reached for 106 deaths and 25 were coded as missing data because of insufficient clinical detail for the reviewers to agree. Allergen exposure data were other allergen or unknown for 20 participants. There were no deaths from allergy immunotherapy or allergen skin prick testing. The overall rate of pre‐existing asthma/COPD was 48.6%, and in the food allergy group, it was 90.7%. ASA scores were higher for medication and radiocontrast groups with ASA 3+ 58% and 66%, respectively.

### 3.1 Primary Outcome

The primary organ failure causing cardiorespiratory decompensation was able to be recorded in 250 (67%) cases. Of those with available data, the most common primary organ failure in all cases was bronchospasm (*n* = 153, 61%), followed by cardiovascular (*n* = 73, 29%) and upper airway obstruction (*n* = 24, 10%) (Table 2). Respiratory failure as a composite of either bronchospasm or upper airway obstruction was responsible for 71% of anaphylaxis deaths. The effect of allergen trigger category is presented with adjusted OR and CI in Table 3. Critically, in food‐related anaphylaxis deaths, observations for the primary organ failure were complete in 58/64 (91%) of reported fatalities. Of these, bronchospasm (asthma) was the primary mode of organ failure in 55 (95%). This is in contrast to deaths due to insect venom allergy where cardiovascular failure was the primary mode of organ failure in 21 (57%), albeit with missing observations in 26 (41%).

**TABLE 2:** Organ failure by allergen exposure.

	Overall sample*	Allergen exposure			
		Drug	Insect venom	Food	Radiocontrast
Number of eligible patients	*N* = 371	*N* = 179	*N* = 63	*N* = 64	*N* = 45
Primary organ failure					
Upper airway	24 (9.6%)	7 (6.1%)	7 (18.9%)	NR	NR
Bronchospasm	153 (61.2%)	68 (59.1%)	9 (24.3%)	55 (94.8%)	13 (48.1%)
Cardiovascular	73 (29.2%)	40 (34.8%)	21 (56.8%)	0 (0.0%)	12 (44.4%)
Missing observations	121	64	26	6	18 observations
Secondary organ failure					
Upper Airway	37 (36.3%)	13 (25.0%)	8 (47.1%)	14 (70.0%)	NR
Bronchospasm	13 (12.7%)	7 (13.5%)	NR	0 (0.0%)	NR
Cardiovascular	52 (51.0%)	32 (61.5%)	5 (29.4%)	6 (30.0%)	9 (69.2%)
Missing observations	249	127	46	44	

* Overall sample includes primary outcome for missing/other allergens and counts otherwise NR in allergen exposure categories. NR, not reportable (Counts less than five are not directly reported as a condition of the Victorian Department of Justice Human Ethics Approval).

**TABLE 3:** Effect of allergen trigger on primary organ failure.

Exposure or contrast	Number (%) with bronchospasm as primary organ failure		Number (%) with cardiovascular as primary organ failure		Number (%) with upper airway obstruction as primary organ failure	
Drug	68/115 (59.1%)		40/115 (34.8%)		7/115 (6.1%)	
Insect venom	9/37 (24.3%)		21/37 (56.8%)		7/37 (18.9%)	
Food	55/58 (94.8%)		0/58 (0.0%)		NR	
Radiocontrast	13/27 (48.1%)		12/27 (44.4%)		NR	

	Odds ratio (95% CI)*	*p*	Odds ratio (95% CI)*	*p*	Odds ratio (95% CI)**	*p*
Insect venom vs drug	0.185 (0.073 to 0.466)	< 0.001	3.076 (1.296 to 7.305)	0.011	3.610 (1.186 to 10.991)	0.024
Food vs drug	2.867 (0.641 to 12.827)	0.167	Cannot be estimated	N/A	2.113 (0.277 to 16.150)	0.468
Radiocontrast vs drug	0.445 (0.168 to 1.175)	0.101	1.503 (0.612 to 3.691)	0.371	2.565 (0.566 to 11.621)	0.220
Food vs insect venom	15.530 (3.166 to 76.186)	0.001	Cannot be estimated	N/A	0.585 (0.079 to 4.343)	0.598
Radiocontrast vs insect venom	2.410 (0.648 to 8.964)	0.187	0.489 (0.157 to 1.516)	0.213	0.711 (0.131 to 3.844)	0.690
Radiocontrast vs food	0.155 (0.027 to 0.901)	0.038	Cannot be estimated	N/A	1.214 (0.098 to 15.087)	0.879

*Note:* All analyses used multiple imputation to handle missing data. Complete analysis vs. multiple imputation can be found in Table S2. NR, not reportable (Counts less than 5 are not directly reported as a condition of the Victorian Department of Justice Human Ethics Approval).

* Analysis model adjusted for age and use of antihypertensives (both effect modifiers), and sex and ASA.

** Analysis model adjusted for age (effect modifier), sex and ASA.

Statistical analysis confirmed these strong clinically meaningful differences in outcomes between allergen exposure groups. The odds of bronchospasm in food exposures compared to insect triggers was very high (OR = 15.5, 95% CI 3.2 to 76.2, *p* = 0.001). Those with insect venom exposures had lower odds of bronchospasm being the primary organ failure compared to drug exposure (OR = 0.2, 95% CI 0.1 to 0.5, *p* < 0.001) as did those with radiocontrast exposure compared to food (OR = 0.2, 95% CI 0.03 to 0.9, *p* = 0.04).

Insect venom exposure was more likely to result in primary cardiovascular failure when compared to drug exposures. For primary upper airway obstruction leading to cardiorespiratory arrest, there was generally little difference in the odds of this outcome between allergen exposures.

In those under 40 years of age, primary organ failure was bronchospasm 90%, cardiovascular 8% and upper airway 2%. In those 40 years or older, the percentages of primary organ failure were bronchospasm 52%, cardiovascular 38%, upper airway 9.7%.

### 3.2 Medication Deaths

There were 179 medication deaths comprising 88 perioperative deaths and 91 deaths outside the operating theatre/recovery environment. Perioperative allergen triggers were neuromuscular blocking agents (*n* = 51, 65%), beta lactam antibiotics (*n* = 17, 22%), other drugs (*n* = 10, 13%), and in nine cases, the exact drug could not be determined. The primary organ failure was bronchospasm (*n* = 33, 54%), cardiovascular (*n* = 28, 46%) with missing data in 27. In the 91 medication deaths outside the perioperative environment, 52 (64%) occurred in the acute hospital setting and 22 (27%) occurred at home with seven occurring in other clinical settings where healthcare professionals were present. Twenty‐five deaths were from oral medication, and in 52, the allergen was injected intravenously. Primary organ failure for the non‐anaesthetic medication group was combined upper and lower airway (*n* = 35, 75%), cardiovascular (*n* = 12, 25%) with missing data in 34. With respect to age and organ failure, 90% (9/10) drug‐related anaphylaxis deaths, including perioperative cases, under the age of 40 were secondary to bronchospasm.

### 3.3 Insect Venom Deaths

There were 63 insect anaphylaxis deaths from exposure to bee sting (*n* = 45, 71%), with the remainder mostly due to wasp sting (*n* = 9, 14%) or myrmecia ant sting (*n* = 7, 11%). Almost all insect venom exposures were in the outside environment, and 52% at their own property. Primary organ failure was recorded in only 59% of deaths because there was frequently rapid or unwitnessed deterioration with no information recording the clinical sequence of events prior to arrest. Adrenaline was administered in only 9 (16%) cases prior to cardiorespiratory arrest, with 8 cases missing data.

### 3.4 Food Deaths

There were 64 food anaphylaxis deaths with major food allergens seafood (*n* = 20, 31%), tree nuts (*n* = 11, 17%), peanut (*n* = 11, 17%), cow's milk (*n* = 8, 13%) and 13 (20%) where the exact allergen could not be determined. Food anaphylaxis was wholly a respiratory death, with bronchospasm the primary organ failure in 95%. Upper airway oedema was present in 22 (43.1%) but was rarely deemed to be the primary cause of arrest. Adrenaline, the majority from patient autoinjectors, was administered intramuscularly in 27 (52%) who progressed to respiratory arrest despite this therapy; 25 (48%) did not receive adrenaline prior to arrest and 12 had missing data. Intubation occurred in 50 (83.3%, four missing data), all after cardiorespiratory arrest. Six patients had a difficult airway during intubation. Vomiting and aspiration occurred in 26 (40.6%), which was substantially higher than other triggers with an OR of 5.5 (95% CI 2.9–10.1, *p* < 0.001).

### 3.5 Time Sequence of Fatal Anaphylaxis

The time sequence of fatal anaphylaxis cases is presented in Table 4, specifically time to severe symptoms from symptom onset, followed by the time interval from severe symptoms to cardiac arrest. All drug cases are included in the analysis as planned, but in a subgroup analysis of oral drugs alone, the median time to severe symptoms from onset was 5.0 min (IQR 5.0‐10) and time to arrest from severe symptoms was 14 min (IQR 10‐20), longer than the parenteral route.

**TABLE 4:** Time sequence of fatal anaphylaxis symptoms.

Exposure or contrast (N)	Median (IQR) time to severe symptoms* from symptom onset in minutes		Median (IQR) time to cardiac arrest from severe symptom onset in minutes	
Drug (112)	1 (1 to 5)		1.2 (1 to 5)	
Insect venom (32)	5 (3 to 10)		5 (2 to 10)	
Food (53)	20 (15 to 30)		10 (10 to 15)	
Radiocontrast (28)	2 (1 to 5)		2 (1 to 4)	

	Median difference (95% CI)*	*p*	Median difference (95% CI)**	*p*
Insect venom vs Drug	3.220 (0.840 to 5.600)	0.008	3.010 (1.144 to 4.876)	0.002
Food vs Drug	17.480 (12.857 to 22.103)	< 0.001	8.010 (6.174 to 9.846)	< 0.001
Radiocontrast vs Drug	0.540 (−1.925 to 3.005)	0.666	0.170 (−2.039 to 2.379)	0.880
Food vs Insect venom	14.260 (9.679 to 18.841)	< 0.001	5.000 (2.812 to 7.188)	< 0.001
Radiocontrast vs Insect venom	−2.680 (−5.485 to 0.125)	0.061	−2.840 (−5.351 to −0.329)	0.027
Radiocontrast vs Food	−16.940 (−22.027 to −11.853)	< 0.001	−7.840 (−10.328 to −5.352)	< 0.001

* Severe symptom definition was determined for this study by the investigators to correlate with hypoxia and hypotension and is based on a combination of Grade 3 symptoms published by Brown [4], Grade 3 features according to the Australian New Zealand College of Anaesthetists [13, 14] and physiological criteria in paediatric age‐specific hospital‐based rapid response criteria [15]. Full criteria are contained in the Supporting Information.

** Analysis used multiple imputation to handle missing data.

### 3.6 Complications and Interventions

There were 27 difficult airways including 12 cricothyroidotomies occurring in all allergen groups except radiocontrast and was significant for insect venom exposure. There were 27 deaths with pneumothoraxes, 14 of which were unilateral and 13 bilateral. Vomiting and aspiration was significant for the food (*n* = 26, 40.6%, *p* = < 0.001) and drug (*n* = 19, 10.6%, *p* = 0.003) groups. Cardioversion for a shockable rhythm was recorded in 51 patients. Association between complications and primary outcome is published in Table S3 and association between complications and allergen exposure is published in Table S4.

## 4 Discussion

This is the first study in our knowledge to rigorously determine and report the type of organ failure leading to cardiorespiratory arrest in anaphylaxis fatalities using a population‐based cohort. Three major findings arise that have significance for international anaphylaxis management and resuscitation guidance. First, we found that respiratory failure rather than cardiovascular failure was the most common cause of cardiorespiratory arrest in fatal anaphylaxis. Second, we found that there are distinct differences in the patterns of organ system failure and timing observed with different allergen triggers. Third, food anaphylaxis deaths were unique in that they were exclusively respiratory in nature with 95% due to bronchospasm.

There was a high burden of respiratory disease in the study cohort, which may reflect that those with reactive airways are predisposed to bronchospasm and therefore are at higher risk of this type of organ failure. Similarly, those who are younger (< 40 years) had very low rates of cardiovascular failure causing death, possibly reflecting their higher cardiovascular reserve compared to the older group where cardiovascular failure was more common (but still less common than bronchospasm).

Traditionally the cardiovascular component of anaphylaxis has been prioritised in treatment guidelines that emphasise linked interventions including intravenous fluids and vasopressors [16, 17, 18]. Indeed sudden hypotension is common in perioperative drug and insect venom anaphylaxis [19, 20], which is supported by our data, and highlights the need for immediate adrenaline in these cases. However, our findings suggest that bronchospasm (asthma, lower respiratory obstruction) is the most common cause of cardiorespiratory arrest in fatal anaphylaxis, especially when triggered by food and drugs. Our data highlight the need for bronchospasm to be appropriately emphasised in anaphylaxis treatment guidelines with respect to recognition and treatment with adrenaline as the first line therapy. The institution of other asthma bronchodilator therapies for lower airway obstruction as concomitant therapy, in addition to adrenaline, may be underemphasised in current guidelines, as may the role of respiratory support in treating fulminant bronchospasm. We hypothesise that extrapolating accepted acute asthma therapies to management of acute bronchospasm in anaphylaxis may be beneficial when administered together with adrenaline to reduce the risk of death.

Unlike cardiovascular failure, which relies on vascular access for fluid and vasopressor administration, fulminant bronchospasm leading to unconsciousness requires bronchodilator administration (adrenaline), securing the airway with early endotracheal intubation and positive pressure ventilation [21]. The complexities of delivering these therapies may be one reason that respiratory failure is more common in anaphylaxis deaths. We hypothesise that other reasons may include that people with severe bronchospasm compensate until sudden respiratory arrest, that severity may be underappreciated by both patients and clinicians during the early compensatory phase, and that bronchospasm may be more likely to be refractory to initial adrenaline.

Treatment of fulminant bronchospasm leading to unconsciousness and patient deoxygenation requires oxygen to be delivered at high peak pressures (well above 30cmH_2_O) into the trachea to overcome bronchiolar resistance. The accepted method of delivering oxygen most reliably in this circumstance is tracheal intubation and ventilation with permissive hypercapnia to prevent barotrauma [22, 23, 24]. Much lower pressures are generated within the respiratory tree by bag‐valve‐mask (BVM) ventilation and supraglottic airway devices (SGAs), limited by air leaking along the path of least resistance into the oesophagus or external environment making them unlikely to be effective [25, 26, 27]. Emphasis on early tracheal intubation by trained health professionals as definitive care to achieve oxygenation in those patients who are decompensating, unconscious or in cardiac arrest from hypoxia may be necessary to reduce the risk of death in this group of patients [22, 28].

Our data suggest that there may be opportunity for further reducing food‐related anaphylaxis deaths. There appears to be time from symptom onset to severe symptoms to call for help and administer intramuscular adrenaline early. Even though this period is short, outcomes may be better if patients and clinicians are educated regarding the significance of respiratory symptoms in food‐triggered anaphylaxis, particularly in patients with co‐existing asthma. Half of food allergy deaths were refractory to initial intramuscular adrenaline, confirming multiple doses may be required for refractory bronchospasm and the importance of calling for help to escalate care. Once severe symptoms are present (hypoxia), it is important that clinicians are aware of the potential rapidity of deterioration to cardiorespiratory arrest (median 10 min, IQR 10‐15) and that they have a short but significant period of time to abort or prepare for this event. Upper airway oedema is a common coexisting feature in food deaths, which has implications for airway management. Vomiting and aspiration, a catastrophic event that worsens bronchospasm, is common in food deaths, suggesting unconscious patients should have their airway rapidly and definitively secured for multiple indications. Despite this, the majority of patients did not classify as having a difficult airway on intubation, suggesting that endotracheal intubation is possible in most cases.

The results of this study are limited by its retrospective design and the poor recording of some treatments like time to intubation. This is an appropriate method for a rare and unpredictable outcome; however, this may lead to a potentially important amount of missing data for the primary outcome (32.6%), introducing selection bias. However, we have attempted to mitigate this by using multiple imputation to handle the missing data under plausible missingness mechanism assumptions.

While this study can be considered reflective of a whole population, there are likely to be cases of anaphylaxis that are not reported to the coroner in Australia and therefore not contained within the NCIS. This occurs where circumstances and cause of death are clear and the requirement for notification of an unexpected death is interpreted differently by individual medical practitioners. In a previous anaphylaxis study comparing national death certificates through the Australian Bureau of statistics and NCIS case reports between 2000 and 2013, 223 of 298 were recorded in the NCIS [10]. It is not possible to determine which cases are not reported to state coroners or whether non NCIS ABS death certificates accurately record anaphylaxis as a true diagnosis. As an added limitation in measuring a whole population, there were some cases in the NCIS with no reports or data to substantiate an anaphylaxis diagnosis for inclusion.

A strength of this study is that data were extracted using a rigorously designed retrospective cohort study of 371 anaphylaxis deaths, the largest published cohort to date. Our findings, for the first time, provide primary data on organ failure in fatal anaphylaxis with a rigorous predetermined categorisation for data extraction for this outcome.

## Author Contributions

Ben A. McKenzie developed the review protocol, extracted data, analysed results and wrote the first draft. Jo A. Douglass, Lena A. Sanci and Stuart D. Marshall provided expert panel input throughout the project including protocol development, data analysis, draft editing and access to/verification of data. Catriona Moynihan extracted data. All authors contributed to the final manuscript.

## Funding

BM is supported by a PhD Scholarship from the Australian Government funded National Allergy Centre of Excellence (NACE), hosted by the Murdoch Children’s Research Institute (MCRI), and their work was supported by the Victorian Government’s Operational Infrastructure Support Program. The funder had no role in the study design, data collection, data analysis, data interpretation or writing of the report.

## Conflicts of Interest

All authors have completed an ICMJE disclosure form (available on request from the corresponding author) and declare support from the Australian Government funded National Allergy Centre of Excellence for the submitted work; BM’s son died from anaphylaxis; no financial relationships with any organisations that might have an interest in the submitted work in the previous 3 years; no other relationships or activities that could appear to have influenced the submitted work.

## Supporting information


**Table S1:** Organ failure allocation descriptors.
**Table S2a:** Summary of overall average causal effect of allergen exposure on primary organ failure (bronchospasm).
**Table S2b:** Summary of overall average causal effect of allergen exposure on primary organ failure (cardiovascular).
**Table S2c:** Summary of overall average causal effect of allergen exposure on primary organ failure (upper airway).
**Table S3:** Association between complication and primary organ failure.
**Table S4:** Association between complication and allergen exposure.

## Data Availability

The data that support the findings of this study are available from the National Coronial Information System under the auspice of the Victorian Government Department of Justice. Restrictions apply to the availability of these data, which were used under licence for this study. Data are available from the author(s) with the permission of the National Coronial Information System.

## References

[1] T. E. Dribin , H. A. Sampson , C. A. Camargo , et al., “Persistent, Refractory, and Biphasic Anaphylaxis: A Multidisciplinary Delphi Study,” Journal of Allergy and Clinical Immunology 146, no. 5 (2020): 1089–1096. 32853640 10.1016/j.jaci.2020.08.015 PMC8006564

[2] M. Worm , O. Eckermann , S. Dölle , et al., “Triggers and Treatment of Anaphylaxis: An Analysis of 4,000 Cases From Germany, Austria and Switzerland,” Deutsches Ärzteblatt International 111, no. 21 (2014): 367–375. 24939374 10.3238/arztebl.2014.0367 PMC4075276

[3] L. L. Reber , J. D. Hernandez , and S. J. Galli , “The Pathophysiology of Anaphylaxis,” Journal of Allergy and Clinical Immunology 140, no. 2 (2017): 335–348. 28780941 10.1016/j.jaci.2017.06.003 PMC5657389

[4] S. G. Brown , “Clinical Features and Severity Grading of Anaphylaxis,” Journal of Allergy and Clinical Immunology 114, no. 2 (2004): 371–376. 15316518 10.1016/j.jaci.2004.04.029

[5] T. E. Dribin , D. Schnadower , J. M. Spergel , et al., “Severity Grading System for Acute Allergic Reactions: A Multidisciplinary Delphi Study,” Journal of Allergy and Clinical Immunology 148, no. 1 (2021): 173–181. 33476673 10.1016/j.jaci.2021.01.003 PMC8273088

[6] S. Perez‐Codesido , A. Rosado‐Ingelmo , M. Privitera‐Torres , et al., “Incidence of Fatal Anaphylaxis: A Systematic Review of Observational Studies,” Journal of Investigational Allergology & Clinical Immunology 32, no. 4 (2022): 245–260. 33856349 10.18176/jiaci.0693

[7] R. Pumphrey , “Lessons for Management of Anaphylaxis From a Study of Fatal Reactions,” Clincal and Experimental Immunology 30 (2000): 1144. 10.1046/j.1365-2222.2000.00864.x 10931122

[8] Y. S. Xu , M. Kastner , L. Harada , A. Xu , J. Salter , and S. Waserman , “Anaphylaxis‐Related Deaths in Ontario: A Retrospective Review of Cases From 1986 to 2011,” Allergy, Asthma and Clinical Immunology 10, no. 1 (2014): 38. 10.1186/1710-1492-10-38 PMC4322510 25670935

[9] P. Martinez‐Fernandez , G. Vallejo‐de‐Torres , M. S. Sanchez‐de‐Leon‐Robles , et al., “Medical and Pathologic Characteristics of Fatal Anaphylaxis: A Spanish Nationwide 17‐Year Series,” Forensic Science, Medicine, and Pathology 15, no. 3 (2019): 369–381. 31292823 10.1007/s12024-019-00134-1

[10] R. J. Mullins , B. K. Wainstein , E. H. Barnes , W. K. Liew , and D. E. Campbell , “Increases in Anaphylaxis Fatalities in Australia From 1997 to 2013,” Clinical and Experimental Allergy 46, no. 8 (2016): 1099–1110. 27144664 10.1111/cea.12748

[11] E. von Elm , D. G. Altman , M. Egger , S. J. Pocock , P. C. Gøtzsche , and J. P. Vandenbroucke , “The Strengthening the Reporting of Observational Studies in Epidemiology (STROBE) Statement: Guidelines for Reporting Observational Studies,” Lancet 370, no. 9596 (2007): 1453–1457. 18064739 10.1016/S0140-6736(07)61602-X

[12] S. Burgess , I. R. White , M. Resche‐Rigon , and A. M. Wood , “Combining Multiple Imputation and Meta‐Analysis With Individual Participant Data,” Statistics in Medicine 32, no. 26 (2013): 4499–4514. 23703895 10.1002/sim.5844 PMC3963448

[13] H. Kolawole , S. D. Marshall , H. Crilly , R. Kerridge , and P. Roessler , “Australian and New Zealand Anaesthetic Allergy Group/Australian and New Zealand College of Anaesthetists Perioperative Anaphylaxis Management Guidelines,” Anaesthesia and Intensive Care 45, no. 2 (2017): 151–158. 28267936 10.1177/0310057X1704500204

[14] R. Tran , K. Pedersen , H. Kolawole , P. Roessler , and R. Scolaro , “Australian and New Zealand Anaesthetic Allergy Group/Australian and New Zealand College of Anaesthetists Perioperative Anaphylaxis Management Guideline 2022,” Anaesthesia and Intensive Care 52, no. 3 (2024): 147–158. 38587791 10.1177/0310057X231215823

[15] Safer Care Victoria , “Victorian Children's Tool for Observation and Response (ViCTOR). Royal Childrens Hospital Clinical Practice Guidelines,” https://www.rch.org.au/educationhub/Education_resources/ViCTOR/.

[16] J. Ring , K. Beyer , T. Biedermann , et al., “Guideline (S2k) on Acute Therapy and Management of Anaphylaxis: 2021 Update,” Allergo Journal International 30, no. 1 (2021): 1–25. 33527068 10.1007/s40629-020-00158-y PMC7841027

[17] Australian Society of Allergy and Clinical Immunology (ASCIA) , “ASCIA Guidelines Acute Management Anaphylaxis,” https://www.allergy.org.au/hp/papers/acute‐management‐of‐anaphylaxis‐guidelines.

[18] M. McLure , K. Eastwood , M. Parr , and J. Bray , “A Rapid Review of Advanced Life Support Guidelines for Cardiac Arrest Associated With Anaphylaxis,” Resuscitation 159 (2021): 137–149. 33068641 10.1016/j.resuscitation.2020.10.001

[19] N. J. N. Harper , T. M. Cook , T. Garcez , et al., “Anaesthesia, Surgery, and Life‐Threatening Allergic Reactions: Management and Outcomes in the 6th National Audit Project (NAP6),” British Journal of Anaesthesia 121, no. 1 (2018): 172–188. 29935569 10.1016/j.bja.2018.04.015

[20] S. G. Brown , K. E. Blackman , V. Stenlake , and R. J. Heddle , “Insect Sting Anaphylaxis; Prospective Evaluation of Treatment With Intravenous Adrenaline and Volume Resuscitation,” Emergency Medicine Journal 21, no. 2 (2004): 149–154. 14988337 10.1136/emj.2003.009449 PMC1726302

[21] Australian New Zealand Council on Resuscitation Guidelines (ANZCOR) , “Guideline 11.2 Resuscitation in Special Circumstances. 2: Asthma,” https://www.anzcor.org/home/adult‐advanced‐life‐support/guideline‐11‐10‐resuscitation‐in‐special‐circumstances.

[22] D. V. Tuxen and S. Lane , “The Effects of Ventilatory Pattern on Hyperinflation, Airway Pressures, and Circulation in Mechanical Ventilation of Patients With Severe Air‐Flow Obstruction,” American Review of Respiratory Disease 136, no. 4 (1987): 872–879. 3662241 10.1164/ajrccm/136.4.872

[23] G. M. Mutlu , P. Factor , D. E. Schwartz , and J. I. Sznajder , “Severe Status Asthmaticus: Management With Permissive Hypercapnia and Inhalation Anesthesia,” Critical Care Medicine 30, no. 2 (2002): 477–480. 11889333 10.1097/00003246-200202000-00034

[24] J. W. Leatherman , C. McArthur , and R. S. Shapiro , “Effect of Prolongation of Expiratory Time on Dynamic Hyperinflation in Mechanically Ventilated Patients With Severe Asthma,” Critical Care Medicine 32, no. 7 (2004): 1542–1545. 15241099 10.1097/01.ccm.0000130993.43076.20

[25] J. Werner , O. Klementova , J. Bruthans , et al., “Evaluation of the i‐Gel Plus Supraglottic Airway Device in Elective Surgery: A Prospective International Multicentre Study*,” Anaesthesia 79, no. 12 (2024): 1284–1291. 39110995 10.1111/anae.16401

[26] L. Theiler , M. Gutzmann , M. Kleine‐Brueggeney , N. Urwyler , B. Kaempfen , and R. Greif , “I‐Gel Supraglottic Airway in Clinical Practice: A Prospective Observational Multicentre Study,” British Journal of Anaesthesia 109, no. 6 (2012): 990–995. 22956643 10.1093/bja/aes309

[27] American Heart Association , “Part 6: Advanced Cardiovascular Life Support,” Circulation 102 (2000): 95–104.

[28] D. V. Tuxen , T. J. Williams , C. D. Scheinkestel , D. Czarny , and G. Bowes , “Use of a Measurement of Pulmonary Hyperinflation to Control the Level of Mechanical Ventilation in Patients With Acute Severe Asthma,” American Review of Respiratory Disease 146, no. 5 Pt 1 (1992): 1136–1142. 1443862 10.1164/ajrccm/146.5_Pt_1.1136

