# Supplementary material for: Mechanism of Cardiac Arrest in Fatal Anaphylaxis
Source: Clin Exp Allergy. 2026 Mar 24;56(5):506–15. doi: 10.1111/cea.70289 (PMC13135877; doi:10.1111/cea.70289)
Supplement: Supplementary file 1 — Table S1: Organ failure allocation descriptors. Table S2a: Summary of overall average causal effect of allergen exposure on primary organ failure (bronchospasm). Table S2b: Summary of overall average causal effect of allergen exposure on primary organ failure (cardiovascular). Table S2c: Summary of overall average causal effect of allergen exposure on primary organ failure (upper airway). Table S3: Association between complication and primary organ failure. Table S4: Association between complication and allergen exposure. [file CEA-56-506-s001.docx]

**SUPPLEMENTARY MATERIAL**

**Mechanism of cardiac arrest in fatal anaphylaxis**

Ben A McKenzie, MBBS ^a,b,c,e^ Stuart D Marshall, PhD^c^ Prof Lena A Sanci, PhD^d^ Catriona Moynihan, MBBS ^e^ Dr Chris Selman, PhD^c^ Prof Jo A Douglass, MD^a,f^

^a^ Department of Medicine, Melbourne Medical School, University of Melbourne, Melbourne, VIC, Australia

^b^ National Allergy Centre of Excellence (NACE), Parkville, VIC, Australia

^c^ Department of Critical Care, Melbourne Medical School, University of Melbourne, VIC, Australia

^d^ Department of General Practice and Primary Care, Melbourne Medical School, University of Melbourne, VIC, Australia

^e^ Department of Emergency Medicine, Royal Melbourne Hospital, Parkville, VIC, Australia

^f^ Department of Allergy and Immunology, Royal Melbourne Hospital, Parkville, VIC, Australia

Corresponding Author

Dr Ben McKenzie [ben.mckenzie@unimelb.edu.au](mailto:ben.mckenzie@unimelb.edu.au) Department of Medicine, Melbourne Medical School, University of Melbourne, Parkville, VIC 3010. +61408020119

**CONTENTS**

| Table S1 Organ failure allocation descriptors | Page 2 |
| --- | --- |
| Study Protocol | Page 3 |
| Statistical Analysis Plan – Page 26-3 | Page 26 |
| Table S2 - Primary outcome complete analysis vs multiple imputation analysis. | Page 34 |
| Table S3 - Association of complication with Primary Organ Failure | Page 36 |
| Table S4 – Association of complication with allergen exposure category | Page 37 |

**Table S1 - Organ failure descriptors**

| **Upper airway** positive for inclusion and allocation to primary or secondary organ failure by critical care specialists:   - Stridor - Altered voice - Tongue/oropharyngeal swelling - Obvious laryngeal swelling at 1^st^ laryngoscopy - Gross macroscopic laryngeal swelling at autopsy - Recorded as Negative/No Upper airway involvement if only microscopic/minor laryngeal swelling at autopsy.   Notes: Criteria developed on expert consensus with consideration of upper respiratory descriptors in published grading systems. Laryngeal swelling may occur after multiple laryngoscopy attempts^1^ – first laryngoscopy and autopsy findings have been qualified to take this confounding element into consideration. Number of laryngoscopy attempts is recorded during data extraction. |
| --- |
| **Bronchospasm** positive for inclusion and allocation to primary or secondary organ failure by critical care specialists:   - Persistent cough - Wheeze - Chest tightness with history of asthma - Shortness of breath with history of asthma - Cyanosis or hypoxia <93% with history of asthma (excluding isolated airway obstruction) - Cyanosis hypoxia <93% without documented or clinical hypotension - Increased work of breathing (excluding isolated upper airway obstruction) - Patient’s usual asthma symptoms - Bronchodilator use during episode - High peak inspiratory pressure in intubated patient (subjective or greater than 30cm H2O) - Difficult to ventilate - Upsloping CO2 trace or obstructive airway pattern   Notes: Criteria developed on expert consensus with consideration of lower respiratory descriptors in published grading systems. Airway obstruction, bronchospasm and cardiovascular symptoms can overlap. Criteria have been developed to exclude overwhelming upper airway obstruction in the absence of obvious bronchospasm. Chest tightness and shortness of breath may be a manifestation of cardiovascular involvement if not associated with asthma symptoms or pre-existing asthma increasing the pre-assessment probability of symptom origin. Dyspnoea, cyanosis and hypoxia can overlap with cardiovascular compromise.^2, 3^ Brown reported 12 % of hypotensive patients developed *dyspnoea* where 100% of hypoxic patients had either dyspnoea or wheeze. In the same study 10% of wheezy patients had hypotension. |
| **Cardiovascular** involvement for inclusion and allocation to primary or secondary organ failure by critical care specialists:   - Hypotension without *severe* respiratory symptoms - Dizziness or feeling faint without *severe* respiratory symptoms - Dyspnoea without severe respiratory symptoms/increase work of breathing. - Postural dizziness without severe respiratory symptoms - Sweating and nausea without severe respiratory symptoms - Tachycardia meeting red ViCTOR^4^ criteria prior to adrenaline and without severe respiratory symptoms - Requiring ongoing vasopressor support even if respiratory symptoms present - Negative/not for inclusion if cardiac arrest only manifestation recorded. |

**Study Protocol**

**Background**

Study of fatal anaphylaxis reactions gives clinicians important information to help avoid this catastrophic outcome in the future, for both patients known to be at risk of anaphylaxis and those who unexpectedly experience a reaction.

Anaphylaxis is an acute-onset, potentially fatal systemic allergic reaction that can affect multiple organs either simultaneously or singularly in isolation. It is usually triggered by an agent such as an insect sting, food, or medication, through a mechanism involving IgE and the high-affinity IgE receptor on mast cells or basophils^5^. Less commonly, it is triggered through other immunologic mechanisms, or through nonimmunologic mechanisms. Anaphylaxis is distinguished from milder allergic reactions when a person’s airway, breathing or circulation become affected^6^. Death occurs when organ failure occurs and is not supported with treatment in the rapid time frame that anaphylaxis evolves within.

Anaphylaxis itself has a spectrum of severity and grading systems exist to quantify the severity of reaction^3, 7-9^. Most cases can be treated with one or two doses of intramuscular adrenaline but approximately 3% of cases need more than two doses^10^. In the most severe reactions, cardiorespiratory arrests occur due to organ failure. These episodes are rare but are an important cause of unexpected and potentially preventable death and disability in all ages^11^. The three main mechanisms of rapid organ failure in anaphylaxis are upper airway oedema, lower airway obstruction principally from bronchospasm, and cardiovascular failure^12^. Beyond treatment with adrenaline, these different types of organ failure require different emergency medical interventions for the patient to survive. Understanding the frequency of each organ system failure in anaphylaxis, including in clinical subgroups, can help inform treatment recommendations to target time critical organ support for these patients.

*Types of organ failure in anaphylaxis*

Anaphylaxis symptoms occur principally through chemical mediators released during mast cell degranulation triggered by cross linking IgE on the mast cell surface by an antigen^13^. A number of chemical mediators are released that include histamine, platelet activating factor, interleukins and leukotrienes. Multiple factors will influence which organs are affected most during an anaphylaxis episode although exact reasons are not well understood. Concentration of antigen and mast cells in a tissue and underlying organ diseases are likely to be significant factors.

*Upper airway oedema*

Mast cells are prevalent throughout the body but have high concentrations in the airway. Histamine and other inflammatory mediators released during anaphylaxis promote vascular permeability and cause swelling. Severe swelling of the tongue, pharynx and larynx can contribute to death through upper airway asphyxia or through impeding delivery of critical medical interventions such as endotracheal intubation which becomes more difficult due to the swelling. Symptoms of airway failure are uncontroversial and hypoxia and cyanosis are late clinical signs prior to death.

*Bronchioles – Bronchospasm – Lower Respiratory Obstruction*

Bronchioles have the highest concentration of mast cells in the airways.^13^ Anaphylaxis inflammatory mediators cause bronchial smooth muscle to contract, promote mucous production and contribute to mucosal wall swelling all causing obstructive airflow limitation. The subsequent increased resistance causes acute hypoxic respiratory failure which can be rapid.

*Cardiovascular*

Inflammatory mediators in anaphylaxis produce vascular vasodilation. Increased vascular permeability causes oedema of body tissues and can cause depletion of intravascular volume contributing to hypotension. Varying degrees of hypotension through this mechanism are described by the widely used Ring and Messner grading scale.

Mast cells also exist in cardiac muscle and their chemical mediators may cause decreased cardiac contractility and may manifest with ECG changes. Mast cells also be part of inflammation in coronary artery atheromatous plaques^14^. Rupture of these plaques during the physiological insult of anaphylaxis has also been reported but may or may not be unique to anaphylaxis.

*Organ failure interrelations and overlap of symptoms*

Vital organ systems do not operate in isolation and are connected in structure and function. There is overlap of symptoms where multiple organ involvement contributes to a clinical endpoint such as hypoxia or hypotension. Cessation of blood flow/absence of blood pressure is the final common pathway but may be caused by hypoxia or primary cardiovascular system compromise or a combination. For example, bronchospasm induced severe hypoxia leads to a bradycardic response and decreased cardiac contractility causing cardiac arrest. Bronchospasm can also induce high intrathoracic pressures decreasing venous return to the heart and cause low blood pressure. At the same time that these respiratory processes are occurring, anaphylaxis mediators may cause systemic vasodilation leading to potentiation of low blood pressure from respiratory causes. A diagram showing interdependencies from an anaesthesia perspective have previously been published.^15^

Similarly reduced cardiac output can result in peripheral hypoxaemia and peripheral cyanosis if severe. It is rare however for central cyanosis to be caused by reduced cardiac output. Of 38 anaphylaxis patients with hypoxia (SaO2 <93%) or cyanosis reported by Brown ^2^, 25 had wheeze, five were deemed to have no wheeze and eight could not be put into either group. Importantly only two were deemed to be due to hypotension alone reinforcing the rarity of cyanosis being caused by the heart and peripheral circulation component of anaphylaxis.

Using a combination of clinical experience and published data, it is feasible to allocate presence or absence of each organ failure type if sufficient clinical details are available. Where there are multiple organs involved it is feasible to record the presence of each organ involved and allocate which is the most significant contributor to death.

*Previously published case series regarding organ failure*

A systematic review studying the type of organ failure in published cases of anaphylaxis identified 14 case series reporting a total of 533 cases. Reporting of organ systems failure leading to death and disability was generally low quality with only one author reporting it as an explicit outcome and this series did not describe the methodology for allocation.

Most series reported respiratory symptoms as a composite of airway oedema and bronchospasm and only three authors separated respiratory components. The predominant organ systems failure or sequence of organ failure was only reported by two authors. Cardiovascular failure ranged from 12% to 100%. Respiratory failure was present in most cases ranging from 53% to 100%. Food related fatalities were children and young adults who died predominantly from respiratory failure with a high frequency of bronchospasm reported (75%). Venom series were older with a very high frequency of cardiovascular collapse (59% to 81%) and lower frequency of respiratory involvement (19% to 53%) which was predominantly upper airway failure when present. Drug related fatalities had high frequencies of both cardiovascular (47% to 100%) and respiratory (60% to 74%) failure.

*Australian National Coronial Information System*

In Australia each state and territory jurisdiction legislates that any unexpected death is reported to the state Coroner, including those that occur in healthcare. The coroner’s role is to establish the cause of death and circumstances through collation of eye witness statements and medical records as well as autopsy. For each reported death the coroner produces formal written findings. The National Coronial Information System contains all deaths reported to state coroners and includes documents that include the formal findings, the police report containing any eyewitness accounts of events surrounding the death, toxicology reports and formal autopsy findings with case synthesis.

Anaphylaxis deaths are all unexpected and are thus reported to state coroners and contained within the NCIS. However, there are some cases where coronial notification is not made and a death certificate is written by medical practitioners without coronial involvement. This occurs where circumstances and cause of death are clear and the requirement for notification is interpreted differently by individual medical practitioners. In a previous anaphylaxis study by Mullins et al comparing national death certificates through the Australian Bureau of statistics and NCIS case reports between 2000 and 2013, 223 out of 298 were recorded in the NCIS.^16^ It is not possible to determine which cases are not reported to state coroners. Despite this missing data, NCIS cases can be considered to be representative of a whole population.

*Cohort Studies*

Cohort studies form a suitable study design to assess associations between multiple exposures on the one hand and multiple outcomes on the other hand. They are especially appropriate to study rare exposures or exposures for which randomization is not possible for practical or ethical reasons^17^, both of which are the case in studying anaphylaxis fatalities. This study will be reported in line with the The Strengthening the Reporting of Observational Studies in Epidemiology (STROBE) statement: guidelines for reporting observational studies^18^.

**Methodology**

*Objective*

To examine differences in organ failure that develop in patients who have fatal anaphylaxis after being exposed to different allergen trigger categories.

*Primary Question*

Is allergen exposure category associated with the type of organ failure that subsequently develops in fatal anaphylaxis?

*Design, Setting and Participants*

This is a population based retrospective analysis of consecutive anaphylaxis deaths of all ages in Australia between 1^st^ January 2003 and 31^st^ December 2022.

*Outcome*

Development of primary organ failure being either due to: Upper airway oedema, Bronchospasm (lower respiratory obstruction), Cardiovascular.

*Exposure*

Patient allergen exposure to one of 4 categories of allergens: Drug; Insect; Food; and Radiology Contrast.

*Covariates*

Covariates collected are Age, pre-existing history of asthma, pre-existing atopic disease, previously allergic to allergen, American Society of Anaesthesia Physical Status score, use of antihypertensives.

*Other variables*

A broad range of variables have been defined (Table 1 and 2) prior to data collection and may be used as covariates – see below

*Ethical Approval*

Approval was obtained from the University of Melbourne Research Ethic Committee, the Victorian Department of Justice Human Research Ethics Committees and the Coroners Court of Victoria Research Committee.

**Data Collection and Management**

*NCIS Case identification*

The NCIS allows for identification of anaphylaxis cases through 3 fields: cause of death free text fields; ICD 10 (coded externally through linkage to the Australian Bureau of Statistics); search of keywords in attached reports. The search strategy for this study is:

1. Cause of death field search using search term “anaphyl*” in all primary and antecedent causes of death listed
2. ICD 10 search
   1. T78 Anaphylaxis (includes food)
   2. T805 Serum Anaphylaxis
   3. T886 Anaphylaxis to drug correctly administered
   4. T634 Arthropod Anaphylaxis
   5. Y575 Drug anaphylaxis
3. Keyword search function of reports
   1. Anaphyl*

We will not separately screen asthma cases for anaphylaxis because a previous NCIS study only found 1 case out 1978 asthma deaths did not have anaphylaxis listed as an antecedent cause.^16^

*Case Screening and Inclusion*

Cases will be screened for inclusion by two critical care physicians who are trained in anaphylaxis case identification independently.

Where there is doubt about the diagnosis of anaphylaxis for inclusion expressed by either physician, cases will be referred to a specialist in Allergy and Immunology for final determination regarding inclusion. Cases identified and those excluded with reasons will be collected on an excel spreadsheet.

*Data Collection*

Study data will be collected and managed using REDCap electronic data capture tools hosted at University of Melbourne. REDCap (Research Electronic Data Capture) is a secure, web-based software platform designed to support data capture for research studies.

Data from each case will be extracted by two critical care clinicians independently. Using the REDCap data comparison tool, any differences will be resolved through agreement and where agreement cannot be reached, the case will be discussed with an expert panel incorporating Anaesthesia, Allergy and Immunology and Primary Care who are part of the study group.

*Data Variables*

Identification of Organ Failure

Instructions for allocation of organ failure were derived from agreement of the expert panel of authors incorporating the specialties of Anaesthesia, Emergency Medicine, Allergy and Clinical Immunology, and Primary Care. The panel used a three step process to develop descriptors of organ involvement. The first was mapping descriptors from nine grading systems identifying symptoms of anaphylaxis and two organ specific (asthma and laryngotracheobronchitis severity descriptors) to categorise them as signifying upper airway, lower airway (bronchospasm) and cardiovascular compromise. The second step involved refining criteria with regard to potential overlap of symptoms/signs including consideration of published data as described above in the background section on organ failure interrelation and overlap of symptoms. The third step was revision from individual domains of expertise from each specialty.

The nine acute allergy grading systems and two organ specific severity guides that covered both adult and paediatric anaphylaxis were mapped to extract symptoms of upper airway, lower respiratory obstruction and cardiovascular symptoms to be identified in the immediate lead up to cardiorespiratory arrest in each case.

- One international allergist based system^19^
- One Delphi study designed for all setting and triggers^7^
- One retrospective ED observation study 30% insects, 22% drugs, 18% food^3^
- One published proposal for universal grading (expert opinion)^20^
- Two grading systems designed for drugs^9, 21^
- Three grading systems designed for allergist food trials^22-24^
- One grading system for insect anaphylaxis^25^
- Two clinical practice guidelines for other conditions with single organ involvement^26, 27^

Once organ involvement was deemed to present the data extractors were required to use clinical assessment as to which organ failure contributed to deterioration prior to cardiac arrest.

When more than one organ failure type was deemed to be present then the data extractors were required to agree on the organ failure that predominantly led to cardiorespiratory arrest. Secondary organ failure was also recorded. The American Society of Anaesthesiologists (ASA) Physical Status Classification System^16^ was used to estimate comorbidities for each case. Other variables collected include age, sex, allergen trigger, pre-existing asthma status, physical location of reaction in relation to healthcare, time from allergen exposure to symptoms, time to severe symptoms, time to cardiorespiratory arrest, whether the patient was administered adrenaline before collapse, intubation status, presence of vomiting or aspiration clinically or on autopsy, whether the patient survived initial resuscitation longer than 2 hours, and the number of days to death if initial survival occurred.

Table 1 contains the data definitions and derivation for complex variables requiring clinical interpretation.

**Table 1 – Data Variable reporting guide for complex variables.**

**1.1 Severe Symptoms, time to.**

| **Definition** | Severe symptoms of anaphylaxis present | | |
| --- | --- | --- | --- |
| **Relevant data fields** | Location where symptoms became severe**;** Was adrenaline given before severe symptoms; Was adrenaline given for severe symptoms before arrest; time from severe symptoms to arrest | **Collection** | Manual |
| **Data Type** |  | **Form** |  |
| **Field Size** |  | **Date started** | 01/05/2024 |
| **Code Sets** | [severe_symptoms_place_diff]  adren_dose_severe  [adrenaline_before_severe] |  |  |
| **Reporting guide** | Severe symptoms are:   - SaO2 < 93% or symptoms associated with hypoxia: - Vomiting, stridor, cyanosis, confusion, dysnoea and wheeze with increased effort.   **OR**   - Hypotension or symptoms associated with hypotension - Vomiting diarrhoea, feeling of impending doom, weakness, dizziness, visual disturbance, diaphoresis, pallor, collapse, incontinence.   **OR**   - Meets clinical review triggers on ViCTOR charts     Please note only one associated symptom required. Some associated symptoms are associated with both hypotension and hypoxia. | | |
| **Purpose and basis for reporting guide** | All patients in this study are deceased and have the most severe grade of anaphylaxis. However deterioration occurs after experiencing less severe grades of anaphylaxis (within varied time frames).  Severe symptoms in this study represent a clinical state occurring prior to arrest. It is an opportunity to examine treatment and situational factors before the patient ultimately enters the peri-cardiorespiratory arrest state. They represent symptoms and signs where there is objective evidence of severe disease and the patient is aware that they need urgent help.  Reporting guidance is based on Grade 3 features developed by Brown^3^ and symptoms statistically significant for hypotension or hypoxia documented in the same analysis. The latter are included to allow for lay reports (eg police reports) to be analysed systematically. Grade 3 Australian New Zealand College Anaesthethetists^21, 28^ and Rapid Review Team/MET criteria represented in the Victorian Childrens Tool for Observation and Response (VICTOR) are also incorporated (eg clinical review triggers)^4^.  Exact times recorded where available. Estimated times recorded where possible to nearest minute for 1-4 minutes, nearest likely 5 minutes for 5-30minutes, nearest 10 minutes greater than 30 minutes. | | |
| **Outcome #** | **Outcome Text Label** | **Outcome Text Label** | **Definition Note** |
| N/A |  |  |  |
|  |  |  |  |

- 1. **Arrest – time to**

| **Definition** | Cardiorespiratory arrest prior to death, or prior to hypoxic brain injury leading to death. | | |
| --- | --- | --- | --- |
| **Field Size** |  | **Date started** | 01/05/2024 |
| **Reporting guide** | Cardiorespiratory arrest = Arrest  Arrest occurs when there is   - Sudden bradycardia - Loss of pulse or BP <50 - Unconscious with ineffective breathing needing assisted ventilation - Seizure or myoclonic activity (hypoxic/hypotensive) - Shockable rhythm/asystole   Exact times recorded where available. Estimated times recorded where possible to nearest minute for 1-4 minutes, nearest likely 5 minutes for 5-30minutes, nearest 10 minutes greater than 30 minutes. | | |
| **Purpose and basis for reporting guide** | To define cardiorespiratory arrest for reference to within study data fields listed above.  There is no universal definition for respiratory arrest and this definition is based on expert opinion for this context.  NAP 6/Anaesthetic threshold for CPR of BP <50 in anaphylaxis incorporated.^29^ | | |
| **Outcome #** | **Outcome Text Label** | **Outcome Text Label** | **Definition Note** |
| N/A |  |  |  |
|  |  |  |  |

**ORGAN FAILIRE TYPES**

- 1. **Upper Airway Oedema present**

| **Definition** | Upper Airway Oedema present during the anaphylaxis episode | | |
| --- | --- | --- | --- |
| **Data name** | Airway Oedema | **Collection** | Manual extraction |
| **Data Type** | Single answer | **Date Started** | 01/05/2024 |
| **Field Size** | Drop down yes/no/not recorded | **Layout** |  |
| **Code Set** | [airway_oedema] = '1' |  |  |
| **Reporting guide** | Yes if ANY of the following present   - Stridor - Altered voice - Tongue/oropharyngeal swelling - Obvious laryngeal swelling at 1^st^ laryngoscopy - Gross macroscopic laryngeal swelling at autopsy | | |
| **Purpose and basis for reporting guide** | To objectively determine presence of upper airway oedema during the anaphylactic reaction that contributed to death.  Criteria developed on expert consensus with consideration of upper respiratory descriptors in published grading systems^3, 7, 21, 30^. Laryngeal swelling may occur after multiple laryngoscopy attempts^1^ – first laryngoscopy and autopsy findings have been qualified to take this confounding element into consideration. Number of laryngoscopy attempts is recorded during data extraction. | | |
| **Outcome #** | **Outcome Text Label** | **Outcome Code Set** | **Definition Note** |
| 1 | Yes |  |  |
| 2 | No |  |  |
| 3 | Not recorded |  | Presence **OR** Absence of symptoms **not** recorded |

- 1. **Bronchospasm present**

| **Definition** | Bronchospasm present during anaphylaxis episode | | |
| --- | --- | --- | --- |
| **Data name** |  | **Collection** | Manual extraction |
| **Data Type** |  | **Date Started** | 01/05/2024 |
| **Field Size** | Drop down yes/no/not recorded | **Layout** |  |
| **Code Set** | [bronchospasm] = 1 |  |  |
| **Reporting guide** | Yes if ANY of the following present   - Persistent cough - Wheeze - Chest tightness with history of asthma - Dyspnoea with history of asthma - Cyanosis or hypoxia <93% with history of asthma (excluding isolated upper airway obstruction) - Cyanosis hypoxia <93% without documented or clinical hypotension - Increased work of breathing (excluding isolated upper airway obstruction) - Patient’s usual asthma symptoms - Bronchodilator use during episode - High peak inspiratory pressure in intubated patient (subjective or greater than 30cm H2O) - Difficult to ventilate - Upsloping CO2 trace or obstructive airway pattern | | |
| **Purpose and basis for reporting guide** | To objectively determine the presence of bronchospasm during the anaphylactic reaction contributing to death.  Criteria developed on expert consensus with consideration of lower respiratory descriptors in published grading systems (^3, 20, 23^3, 6, 7).  Upper airway obstruction, bronchospasm and cardiovascular symptoms can overlap.  Criteria have been developed to exclude overwhelming upper airway obstruction in the absence of obvious bronchospasm. Chest tightness and shortness of breath may be a manifestation of cardiovascular involvement if not associated with asthma symptoms or pre-existing asthma increasing the pre-assessment probability of symptom origin.  Dyspnoea, cyanosis and Hypoxia can overlap with cardiovascular compromise^2, 3^. Brown reported 12 % of hypotensive patients developed *dyspnoea* where 100% of hypoxic patients had either dyspnoea or wheeze. In the same study 10% of wheezy patients had hypotension. | | |
| **Outcome #** | **Outcome Text Label** | **Outcome Code Set** | **Definition Note** |
| 1 | Yes |  |  |
| 2 | No |  |  |
| 3 | Not recorded |  | Presence **OR** Absence of symptoms **not** recorded |

- 1. **Cardiovascular reaction present**

| **Definition** | Cardiovascular reaction present during the anaphylaxis episode | | |
| --- | --- | --- | --- |
| **Data name** | Cardiovascular | **Collection** | Manual extraction |
| **Data Type** | Single answer | **Date Started** | 01/05/2024 |
| **Field Size** | Drop down yes/no/not recorded | **Layout** |  |
| **Code Set** | [cardiovascular] = '1' |  |  |
| **Reporting guide** | Yes if ANY of the following present   - Hypotension without severe respiratory symptoms - Dizziness or feeling faint without severe respiratory symptoms - Dyspnoea without severe respiratory symptoms - Postural dizziness without severe respiratory symptoms - Sweating and nausea without severe respiratory symptoms - Tachycardia meeting red ViCTOR criteria prior to adrenaline and without severe respiratory symptoms - Requiring ongoing vasopressor support even if respiratory symptoms present   Do not say YES if only evidence of cardiovascular reaction is arrest. | | |
| **Purpose and basis for reporting guide** | To objectively determine presence of cardiovascular reaction presence during the anaphylactic episode.  Criteria developed on expert consensus with consideration of cardiovascular descriptors in published anaphylaxis grading scales.  Chest tightness and shortness of breath may be a manifestation of cardiovascular involvement if not associated with asthma symptoms or pre-existing asthma increasing the pre-assessment probability of symptom origin. Hypotensive reactions can overlap symptomatology with severe respiratory symptoms. Brown reported 12 % of hypotensive patients developed *dyspnoea* where 100% of hypoxic patients had either dyspnoea or wheeze. In the same study 10% of wheezy patients had hypotension. | | |
| **Outcome #** | **Outcome Text Label** | **Outcome Code Set** | **Definition Note** |
| 1 | Yes |  |  |
| 2 | No |  |  |
| 3 | Not recorded |  | Presence **OR** Absence of symptoms **not** recorded |

- 1. **Age Groups**

| **Definition** | Predefined age groups | | |
| --- | --- | --- | --- |
| **Data name** | Age group | **Collection** | Post data extraction |
| **Data Type** | Numeric | **Date Started** | 01/05/2024 |
| **Field Size** | N/A | **Layout** | Numeric |
| **Code Set** |  |  |  |
| **Reporting guide** | Age Groups (years)  <5  5-17  18-39  40-59  >=60 | | |
| **Purpose and basis for reporting guide** | To have predefined clinically meaningful age groups.  <5 represent preschool children with different airway anatomy  5-17 represent school age children and allow analysis of paediatric population  18-39 young adults  40-59 older adults  >=60 | | |

- 1. **Primary Organ Failure Type**

| **Definition** | The organ failure type that contributed most to the cardiorespiratory arrest. | | |
| --- | --- | --- | --- |
| **Data name** | primary_organ_failure | **Collection** | Manual |
| **Data Type** | Dropdown | **Form** |  |
| **Field Size** | 1-5 see below | **Layout** |  |
| **Reporting guide** | This is the organ failure type that predominates the reaction – specifically which is most severe.  However if two organ failure elements cannot be delineated between primary and secondary, then the organ with symptoms that were most pronounced first in time should be listed as the primary organ failure type.  A cardiorespiratory arrest that occurs with upper airway oedema or bronchospasm present in isolation should NOT have cardiovascular reaction present after the arrest unless there is prolonged requirement for vasopressor support. | | |
| **Purpose and rationale** | To record the predominant organ failure type leading to cardiorespiratory arrest. | | |
| **Outcome #** | **Outcome Text Label** | **Outcome Text Label** | **Definition Note** |
| 1 | Airway Oedema |  | A cardiorespiratory arrest that occurs predominantly from upper airway oedema symptoms |
| 2 | Bronchospasm |  | A cardiorespiratory arrest that occurs predominantly from bronchospasm symptoms |
| 3 | Cardiovascular |  | A cardiorespiratory arrest that occurs predominantly from cardiovascular symptoms |
| 4 | Pulmonary oedema |  | A cardiorespiratory arrest that occurs predominantly from pulmonary oedema symptoms |
| 5 | Unable to determine |  | Insufficient information to comment. |

- 1. **Secondary Organ Failure Type**

| **Definition** | The organ involvement that was present and contributed less to the cardiorespiratory arrest than the primary organ failure type involved. | | |
| --- | --- | --- | --- |
| **Data name** | secondary_organ_failure | **Collection** | Manual |
| **Data Type** | Dropdown | **Form** |  |
| **Field Size** | 1-5 see below | **Layout** |  |
| **Reporting guide** | This is the organ failure type that was present during the anaphylaxis reaction but was not the primary reason for cardiorespiratory arrest; or was the reaction that occurred second in time if considered equal in magnitude to the primary mechanism.  Example – Upper airway oedema that was observed on laryngoscopy.  in a bronchospasm primary organ failure patient | | |
| **Purpose and rationale** | To record the predominant organ failure type leading to cardiorespiratory arrest. | | |
| **Outcome #** | **Outcome Text Label** | **Outcome Text Label** | **Definition Note** |
| 1 | Airway Oedema |  | A cardiorespiratory arrest that occurs predominantly from upper airway oedema symptoms |
| 2 | Bronchospasm |  | A cardiorespiratory arrest that occurs predominantly from bronchospasm symptoms |
| 3 | Cardiovascular |  | A cardiorespiratory arrest that occurs predominantly from cardiovascular symptoms |
| 4 | Pulmonary oedema |  | A cardiorespiratory arrest that occurs predominantly from pulmonary oedema symptoms |
| 5 | No secondary organ failure reported |  | No other organ type reported as present |

- 1. **Third Organ Failure Type**

| **Definition** | The organ failure type that was present and contributed least to the cardiorespiratory arrest after the primary organ failure type involved. | | |
| --- | --- | --- | --- |
| **Data name** | secondary_organ_failure | **Collection** | Manual |
| **Data Type** | Dropdown | **Form** |  |
| **Field Size** | 1-5 see below | **Layout** |  |
| **Code Set** |  |  |  |
| **Reporting guide** | This is the organ failure type that was present during the anaphylaxis reaction but contributed least to the cardiorespiratory arrest.  Examples 1 - Upper airway oedema on laryngoscopy in a bronchospasm/cardiovascular mixed reaction. | | |
| **Purpose and rationale** | To record the predominant organ failure type leading to cardiorespiratory arrest. | | |
| **Outcome #** | **Outcome Text Label** | **Outcome Text Label** | **Definition Note** |
| 1 | Airway Oedema |  | A cardiorespiratory arrest that occurs predominantly from upper airway oedema symptoms |
| 2 | Bronchospasm |  | A cardiorespiratory arrest that occurs predominantly from bronchospasm symptoms |
| 3 | Cardiovascular |  | A cardiorespiratory arrest that occurs predominantly from cardiovascular symptoms |
| 4 | Pulmonary oedema |  | A cardiorespiratory arrest that occurs predominantly from pulmonary oedema symptoms |
| 5 | No third organ failure type involved |  | No recorded 3^rd^ organ system involved. |

- 1. **Posture Questions**

| **Definition** | What is your opinion regarding the postural collapse? | | |
| --- | --- | --- | --- |
| **Data name** | posture_collapse_opinion | Collection | Manual |
| **Data Type** | Dropdown | **Form** |  |
| **Field Size** | 1-5 see below | **Layout** |  |
| **Code Set** |  |  |  |
| **Reporting guide** | Would the patient have stayed conscious for more than 5 minutes had they not been upright?  Patients who have terminal respiratory physiology are likely to collapse/become unconscious regardless of posture and the impact of decreased venous return is likely to be of modest impact compared to the hypoxia. Cardiovascular reactions are more likely to be impacted by postural decrease in venous return. | | |
| **Purpose and rationale** | Pumphrey^31^ in 2003 published a letter regarding 6 fatalities where the final collapse and subsequent death occurred after a change in posture.  Mullins^16^ looking at the NCIS reported on patients with an upright posture alone without recording a change in posture.  It is unclear whether or not these patients were experiencing a terminal event regardless of posture or whether posture contributed to their death. This data field seeks to clarify the severity of symptoms and whether or not each collapse from upright posture was due to   - the patient reaching the limit of their physiologic compensation regardless of posture. - Posture and decreased venous return contributed to the death. | | |

- 1. **Rebound symptoms – Did symptoms improve at any time?**

| **Definition** | Anaphylaxis symptoms that improve with or without treatment prior to arrest | | |
| --- | --- | --- | --- |
| **Data name** | symptom_rebound | **Collection** | Manual |
| **Data Type** | Yes/No/Not recorded | **Form** |  |
| **Code Sets** |  |  |  |
| **Reporting guide** | Answer YES   - any perceived improvement in symptoms prior to arrest | | |
| **Purpose and rationale** | To record the predominant organ failure type leading to cardiorespiratory arrest. | | |
| **Clarification questions** |  |  | **Definition Note** |
| 1 | How long did symptoms improve for in minutes? |  |  |
| 2 | Did the symptoms improve spontaneously or with adrenaline? |  |  |
| 3 | When symptoms improved, were the improved symptoms severe? |  | See definition of severe symptoms |
| 4 | Did symptoms resolve completely for 1 hour without ongoing treatment before getting worse? |  | Must be completely symptom free including of cutaneous and gastrointestinal symptoms |
| 5 | Notes about rebound symptoms in relation to treatment/times to collapse |  | Describe events and sequence |

- 1. **Complications**

| **Definition** | Anaphylaxis symptoms that improve with or without treatment prior to arrest | | |
| --- | --- | --- | --- |
| **Data name** | symptom_rebound | **Collection** | Manual |
| **Data Type** | Yes/No/Not recorded | **Form** |  |
| **Code Sets** |  |  |  |
| **Reporting guide** | Answer YES   - any perceived improvement in symptoms prior to arrest | | |
| **Purpose and rationale** | To record the predominant organ failure type leading to cardiorespiratory arrest. | | |
| **Clarification questions** |  |  | **Definition Note** |

Table 2 – All data variables

| **Field Type** | **Field Label** | **Choices, Calculations, OR Slider Labels** |
| --- | --- | --- |
| text | NCIS No. |  |
| text | Year |  |
| text | Age |  |
| radio | Gender | 2, Female \| 1, Male |
| checkbox | Is there enough data to continue extraction? | 1, Yes \| 2, No |
| checkbox | Should this case be included as anaphylaxis?  Say no if an alternative diagnosis is more likely | 1, Yes \| 2, No |
| radio | Any history of asthma, atopic, hay fever, prior wheeze or COPD? | 1, Yes \| 2, No \| 3, Not Recorded |
| radio | Any history of atopia (eczema, hayfever, allergies) | 1, Yes \| 2, No \| 3, Not recorded |
| radio | Any history of wheeze/bronchospasm | 1, Yes \| 2, No \| 3, Not recorded |
| radio | Any diagnosis of asthma | 1, Yes \| 2, No \| 3, Not recorded |
| radio | Any diagnosis of COPD | 1, Yes \| 2, No \| 3, Not recorded |
| radio | Uses salbutamol | 1, Yes \| 2, No \| 3, Not recorded |
| radio | uses preventer therapy for asthma or COPD | 1, Yes \| 2, No \| 3, Not recorded |
| radio | Previous hospital admission for asthma ever | 1, Yes \| 2, No \| 3, Not recorded |
| radio | Previous hospital admission in the last 12 months | 1, Yes \| 2, No \| 3, Not recorded |
| dropdown | Asthma/COPD preventer | 1, Alvesco (ciclesonide) \| 2, Flixotide (fluticisone) \| 3, Oxis (eformoterol) \| 4, Pulmicort (budesonide) \| 5, QVAR (beclomethasone) \| 6, Symbicort (budesonide+formoterol) \| 7, Seretide (salmeterol/fluticasone) \| 8, Salmeterol alone \| 9, Flutiform \| 10, Fostair \| 11, Breo Elipta \| 12, Atectura \| 13, Trelegy \| 14, Enerziar |
| text | Asthma/Allergy history notes |  |
| radio | Did the patient have any documented allergies? | 1, Yes \| 2, No \| 3, Not recorded |
| checkbox | Known patient allergens prior to this episode | 35, Cows Milk \| 36, NUTS in General where specific is unknown \| 37, TREE NUTS in general where specific is unknown \| 1, Egg \| 2, Peanuts \| 3, Almonds \| 4, Brazil nuts \| 5, Cashews \| 6, Hazelnuts \| 7, Macadamia nuts \| 8, Pecans \| 9, Pine nuts \| 10, Pistachios \| 11, Walnuts \| 12, Sesame \| 13, Soy \| 14, Wheat \| 15, Crustaceans eg. shrimp, shellfish \| 16, Molluscs eg. mussels, oysters \| 17, Fish \| 20, Ant \| 21, Bee \| 22, Wasp \| 23, Tick \| 24, Penicillin \| 25, Cefalosporin \| 26, Sulfa drugs \| 27, NSAID \| 38, Drug - Other \| 28, Contrast \| 29, Vaccine \| 30, Pollen \| 31, Dust mite \| 32, Animal dander \| 33, Other \| 34, Not recorded |
| text | Other drug(s) specify |  |
| text | Other allergy free text |  |
| radio | Allergist? Had the patient EVER seen an allergist before | 1, Yes \| 2, No \| 3, Not recorded |
| radio | Allergist? Had the patient seen an allergist in the last 5 years? | 1, Yes \| 2, No \| 3, Not recorded |
| radio | Respiratory specialist? Had the patient seen a respiratory specialist in the last 5 years | 1, Yes \| 2, No \| 3, Not recorded |
| radio | Autoinjector script? Was an adrenline autoinjector prescribed for the patient | 1, Yes \| 2, No \| 3, Not recorded |
| radio | Access to own autoinjector? Did the patient carry their autoinjector or have access to their autinjector when symptoms started? | 1, Yes \| 2, No \| 3, Not recorded |
| radio | Allergy Action Plan? Did the patient have an allergy action plan | 1, Yes \| 2, No \| 3, Not recorded |
| radio | Minor allergic reactions? Did the patient have any prior allergic reactions where adrenaline was not required? | 1, Yes \| 2, No \| 3, Not recorded |
| radio | Prior anaphylaxis? Did the patient have any prior allergic reactions where adrenaline was required? | 1, Yes \| 2, No \| 3, Not recorded |
| text | Notes about allergy management/autoinjectors |  |
| checkbox | Patient past medical history and cofactors | 9, Drugs - B Blocker \| 10, Drugs - ACEI \| 11, Drugs - other antihypertensives \| 12, Drugs - NSAID use in last 48 hours \| 14, Intercurrent illness - suspected respiratory virus \| 15, Intercurrent illness - proven respiratory virus \| 16, Intercurrent illness - other \| 17, Intercurrent illness - nil, thought to be well \| 18, Exercise at onset \| 19, Alcohol consumed before onset |
| text | Notes about past medical history/cofactors |  |
| radio | ASA Status  ASA 1  Healthy Patient  ASA2  Mild diseases only without substantive functional limitations. Current smoker, social alcohol drinker, pregnancy, obesity (30<BMI< 40), well-controlled DM/HTN, mild lung , Asymptomatic congenital cardiac disease, well controlled dysrhythmias, asthma without exacerbation, well controlled epilepsy, non-insulin dependent diabetes mellitus, abnormal BMI percentile for age, mild/moderate OSA, oncologic state in remission  ASA3  Substantive functional limitations; One or more moderate to severe diseases. Poorly controlled DM or HTN, COPD, morbid obesity (BMI ≥40), active hepatitis, alcohol dependence or abuse, implanted pacemaker, moderate reduction of ejection fraction, ESRD undergoing regularly scheduled dialysis, history (>3 months) of MI, CVA, TIA, or CAD/stents. Uncorrected stable congenital cardiac abnormality, asthma with exacerbation, poorly controlled epilepsy, insulin dependent diabetes mellitus, morbid obesity, malnutrition, severe OSA, oncologic state, renal failure, muscular dystrophy, cystic fibrosis, history of organ transplantation.  ASA4  Recent (< 3 months) MI, CVA, TIA or CAD/stents, ongoing cardiac ischemia or severe valve dysfunction, severe reduction of ejection fraction, shock, sepsis, DIC, ARD or ESRD not undergoing regularly scheduled dialysis. Symptomatic congenital cardiac abnormality, congestive heart failure, active sequelae of prematurity, acute hypoxic-ischemic encephalopathy, shock, sepsis, disseminated intravascular coagulation, automatic implantable cardioverter-defibrillator, ventilator dependence, endocrinopathy, severe trauma, severe respiratory distress, advanced oncologic state. | 1, 1 \| 2, 2 \| 3, 3 \| 4, 4 |
| radio | Trigger | 1, Food \| 2, Medication \| 4, Immunotherapy/Allergy Testing \| 5, Radiology Contrast \| 6, Venom \| 7, Other substance \| 8, Unable to be determined/idiopathic \| 9, Not recorded |
| radio | Previously diagnosed or documented to be allergic to this allergen? | 1, Yes \| 2, No \| 3, Not recorded |
| text | Unable to be determined/idiopathic - specify details |  |
| text | Other substance specify agent/allergen and route |  |
| radio | Insect Venom Type | 1, Bee \| 2, Wasp \| 3, Ant \| 4, Tick \| 5, Unknown |
| text | Insect further details - eg how many stings, circumstances |  |
| text | Radiology Contrast Specify Agent and Route |  |
| radio | Immunotherapy Route | 1, Subcutaneous (SCIT) \| 2, Sublingual (SLIT) \| 3, Oral \| 4, Topical \| 5, TESTING: SKIN ALLERGEN TESTING ONLY NOT IMMUNOTHERAPY \| 6, TESTING: ALLERGEN CHALLENGE TESTING |
| text | What immunotherapy free text |  |
| radio | Medication category | 1, Neuromuscular blocker \| 2, B-Lactam antibiotic \| 3, NSAID \| 4, Sulfa drug \| 5, Other \| 6, Unknown |
| radio | Medication Route | 1, IV \| 2, IM \| 3, Subcut \| 4, Oral \| 5, Topical/intranasal/inhaled/PR/other route not otherwise listed \| 6, Unknown |
| text | Medication generic name |  |
| radio | Food category | 1, Cows Milk \| 2, Egg \| 3, Peanut \| 4, Tree Nut \| 5, Soy \| 6, Sesame \| 7, Wheat \| 8, Crustaceans eg shrimp shellfish \| 9, Molluscs eg oysters muscles \| 10, Fish \| 11, Unknown - exact food trigger unknown \| 12, Other |
| text | Further food details (eg what food dish) |  |
| checkbox | Food source | 1, Packaged with ingredient label \| 2, Self-sourced \| 3, Catered food \| 4, Restaurant delivered \| 5, Restaurant dine-in \| 6, Given by family - usual household \| 7, Given by family - non usual household \| 8, Given by friends \| 9, Given by Neighbours \| 10, Socially \| 11, Provided by school institution linked activity \| 12, Not recorded |
| text | Food source details eg narrative of food source |  |
| text | Other food free text |  |
| radio | Tree Nut Type | 1, Almonds \| 2, Brazil nuts \| 3, Cashews \| 4, Hazelnuts \| 5, Macadamia nuts \| 6, Pecans \| 7, Pine nuts \| 8, Pistachios \| 9, Walnuts \| 10, Other \| 11, Unknown which tree nut |
| text | Other tree nut free text |  |
| radio | Place of exposure to allergen | 1, Allergy Clinic not part of acute hospital \| 2, Allergy Clinic part of  acute hospital \| 3, Home \| 4, Other friend or family house not home \| 5, School \| 6, Restaurant \| 9, Hospital - Operating theatres/recovery \| 10, Hospital - Acute \| 11, Other healthcare (where health practitioner present) \| 12, Radiology - outside hospital \| 13, Radiology - inside hospital \| 14, Other \| 15, Unknown/Not recorded |
| radio | Patient remote from healthcare? | 1, YES Patient more than 30 minutes drive to urgent care/Emergency Dept/Health post/Ambulance response \| 2, NO  Less than 30 minutes drive to healthcare (eg metropolitan areas or within regional cities towns) |
| text | Place of exposure description free text |  |
| radio | Place where severe symptoms/arrest occurred different to where the patient was exposed?  Severe symptoms are:  - SaO2 < 93% or symptoms associated with hypoxia:  - Vomiting, stridor, cyanosis, confusion, dyspnoea and wheeze with increased effort.  OR  - Hypotension or symptoms associated with hypotension  - Vomiting diarrhoea, feeling of impending doom, weakness, dizziness, visual disturbance, diaphoresis, pallor, collapse, incontinence.  OR  - Meets clinical review triggers on ViCTOR charts | 1, Yes \| 2, No \| 3, Not recorded |
| radio | Location where symptoms became severe | 1, Allergy Clinic not part of acute hospital \| 2, Allergy Clinic part of  acute hospital \| 3, Home \| 4, Other friend or family house not home \| 5, School \| 6, Restaurant \| 9, Hospital - Critical Care Area \| 10, Hospital - Other \| 11, Other healthcare (where health practitioner present) \| 12, Radiology - outside hospital \| 13, Radiology - inside hospital \| 14, Ambulance \| 15, Other \| 16, Unknown/Not recorded |
| text | Severe symptom location description |  |
| text | Minutes from exposure to symptoms: |  |
| text | Minutes from first symptoms to severe symptoms |  |
| text | Minutes from severe symptoms to arrest  Arrest occurs when there is  - Sudden bradycardia  - Loss of pulse or BP <50  - Unconscious with ineffective breathing needing assisted ventilation  - Seizure or myoclonic activity (hypoxic/hypotensive)  - Shockable rhythm/asystole |  |
| text | time from arrest to death |  |
| radio | Airway oedema present | 1, Yes \| 2, No \| 3, Not Recorded |
| text | Evidence of upper airway oedema in a few words/sentence |  |
| radio | Bronchospasm present | 1, Yes \| 2, No \| 3, Not Recorded |
| text | Evidence for bronchospasm in a few words/sentence |  |
| radio | Cardiovascular compromise | 1, Yes \| 2, No \| 3, Not recorded |
| text | Evidence for cardiovascular compromise in a few words/sentence |  |
| dropdown | Primary organ failure | 1, Airway \| 2, Bronchospasm \| 3, Cardiovascular \| 4, unable to determine |
| dropdown | Secondary organ failure | 1, Airway \| 2, Bronchospasm \| 3, Cardiovascular \| 4, No secondary organ failure recorded |
| dropdown | Third Organ Failure | 1, Airway \| 2, Bronchospasm \| 3, Cardiovascular \| 4, No third organ failure recorded |
| checkbox | Clinical features prior to or at the time of collapse/arrest | 1, Vomiting or aspiration at any time, including on autopsy \| 2, Incontinence at any time \| 3, Shortness of breath at any time before collapse/unconsciousness \| 4, Pt states "I'm going to die" before collapse/unconsciousness (screaming for child) \| 12, Pt states "I cant breath" before collapse/unconsciousness \| 5, Impending doom/panic at any time \| 6, Sweating at any time \| 11, Combative \| 10, seizure or myoclonic jerks during collapse \| 9, Bradycardia at anytime \| 7, Cyanosis \| 8, Pallor |
| text | Notes on fatal symptoms |  |
| radio | Posture - Was there a collapse from an upright position (standing or sitting)? | 1, Yes the patient was upright (standing or sitting) WITHOUT change to upright posture in the preceding two minutes \| 2, Yes the patient was upright (standing or sitting) WITH A CHANGE TO UPRIGHT POSTURE in the preceding two minutes \| 3, No the patient was lying down or sitting with legs up \| 4, Not recorded |
| dropdown | What is your opinion regarding the postural collapse? | 1, The patient was in extremis and the agitation/change in posture was part of the terminal event - arrest would have occurred anyway \| 2, The patient would not have arrested had they not had a change in posture at that time. \| 3, Unable to determine either of the above |
| text | Note about your opinion re postural syncope or collapse from upright position |  |
| radio | Did pre-arrest symptoms improve before getting worse? | 1, Yes \| 2, No \| 3, Not recorded |
| text | How long did symptoms improve for in minutes? |  |
| radio | Did the symptoms improve spontaneously or with adrenaline? | 1, Spontaneously \| 2, With adrenaline \| 3, Not recorded |
| dropdown | When symptoms improved, were the improved symptoms severe? | 1, Yes \| 2, No, improved symptoms were not severe \| 3, Not recorded |
| radio | Did symptoms resolve completely for 1 hour without ongoing treatment before getting worse? | 1, Yes \| 2, No \| 3, Not recorded |
| text | Notes about rebound symptoms in relation to treatment/times to collapse |  |
| radio | Was adrenaline given before severe symptoms? | 1, Yes \| 2, No \| 3, Not recorded |
| text | How much IM adrenaline in mg before severe symptoms? |  |
| radio | Was adrenaline given for SEVERE symptoms before arrest? | 1, Yes \| 2, No \| 3, Not Recorded |
| text | How much adrenaline was given for SEVERE symptoms in mg before arrest/unconscious? |  |
| text | Time to IV adrenaline after arrest in minutes |  |
| text | Total dose of adrenaline post arrest |  |
| text | Adrenaline notes |  |
| radio | Was a second line vasopressor/cardiovascular support used (includes usuals and glucagon, methylene blue) | 1, Yes \| 2, No \| 3, Not Recorded |
| radio | Noradrenaline | 1, Yes \| 2, No \| 3, Not Recorded |
| radio | Metaraminol | 1, Yes \| 2, No \| 3, Not Recorded |
| radio | Vasopressin | 1, Yes \| 2, No \| 3, Not Recorded |
| radio | Dopamine | 1, Yes \| 2, No \| 3, Not Recorded |
| radio | Dobutamine | 1, Yes \| 2, No \| 3, Not Recorded |
| radio | Glucagon | 1, Yes \| 2, No \| 3, Not Recorded |
| radio | Methylene Blue | 1, Yes \| 2, No \| 3, Not Recorded |
| radio | milrinone | 1, Yes \| 2, No \| 3, Not Recorded |
| text | Notes about second line cardiovascular support |  |
| Yes/no | Other resuscitation? Did the patient receive resuscitation eg fluids, oxygen, bronchodilators, DCR from any healthcare staff |  |
| radio | Crystalloid use before arrest | 1, Yes \| 2, No \| 3, Not recorded |
| radio | Crystalloid use after arrest | 1, Yes \| 2, No \| 3, Not recorded |
| radio | Salbutamol use | 1, Yes \| 2, No \| 3, Not recorded |
| radio | Ipratropium Use | 1, Yes \| 2, No \| 3, Not recorded |
| radio | Steroid Use | 1, Yes \| 2, No \| 3, Not recorded |
| radio | NIV Use | 1, Yes \| 2, No \| 3, Not recorded |
| radio | Bag and Mask Ventilation only | 1, Yes \| 2, No \| 3, Not recorded |
| radio | FONA front of neck access | 1, Yes \| 2, No \| 3, Not recorded |
| radio | ECMO VV | 1, Yes \| 2, No \| 3, Not recorded |
| radio | ECMO VA | 1, Yes \| 2, No \| 3, Not recorded |
| radio | Intubated | 1, Yes \| 2, No \| 3, Not recorded |
| radio | 1 or 2 shocks required at any time | 1, Yes \| 2, No \| 3, Not recorded |
| radio | 3 or more shocks required at any time | 1, Yes \| 2, No \| 3, Not recorded |
| text | Time from arrest to ECMO in minutes |  |
| dropdown | When was the patient intubated | 1, Intubated before reaction \| 2, Intubated before arrest \| 3, Intubated after arrest |
| dropdown | How many attempts at intubation? | 1, 1 \| 2, 2 \| 3, 3 \| 4, 4 \| 5, 5 \| 6, More than 5 |
| checkbox | Who intubated/attempted to intubate the patient | 1, Paramedic \| 2, ED Physician \| 3, Anaesthetist \| 4, Intensivist \| 5, GP Anaesthetist/generalist |
| text | Minutes to successful definitive intubation from arrest |  |
| text | Free text notes about intubation |  |
| text | Critical Care/Resuscitation Notes |  |
| Yes/no | Was there a pneumothorax? |  |
| Yes/no | Was there any arrhythmia, or shocks delivered |  |
| Yes/no | Was there an AMI, cerebral bleed, DIC, surgical airway complication or any other complication? |  |
| radio | Pneumothorax single lung | 1, Yes \| 2, No \| 3, Not Recorded |
| radio | Pneumothorax both lungs | 1, Yes \| 2, No \| 3, Not Recorded |
| radio | Arrhythmia - VF | 1, Yes \| 2, No \| 3, Not Recorded |
| radio | Arrythmia - VT | 1, Yes \| 2, No \| 3, Not Recorded |
| radio | Arrhythmia - AF | 1, Yes \| 2, No \| 3, Not Recorded |
| radio | Arrhythmia SVT | 1, Yes \| 2, No \| 3, Not Recorded |
| radio | Arrhythmia - Other | 1, Yes \| 2, No \| 3, Not Recorded |
| radio | Acute MI clinically | 1, Yes \| 2, No \| 3, Not Recorded |
| radio | Acute MI - On autopsy | 1, Yes \| 2, No \| 3, Not Recorded |
| radio | Acute MI - Requiring anticoagulation therapy prior to death | 1, Yes \| 2, No \| 3, Not Recorded |
| radio | Intracerebral bleed | 1, Yes \| 2, No \| 3, Not Recorded |
| radio | Complication of surgical airway | 1, Yes \| 2, No \| 3, Not Recorded |
| radio | other serious complication | 1, Yes \| 2, No \| 3, Not Recorded |
| radio | Disseminated Intravascular Coagulation (DIC) | 1, Yes \| 2, No \| 3, Not Recorded |
| radio | Pulmonary oedema at any time | 1, Yes \| 2, No \| 3, Not Recorded |
| radio | Hypoxic brain injury diagnosed before death | 1, Yes \| 2, No \| 3, Not Recorded |
| text | Free text notes about complications |  |

**Data Analysis**

1. Wittekamp BHJ, van Mook WNKA, Tjan DHT, Zwaveling JH, Bergmans DCJJ. Clinical review: Post-extubation laryngeal edema and extubation failure in critically ill adult patients. Critical Care. 2009;13(6):233.

2. Brown SG, Stone SF, Fatovich DM, Burrows SA, Holdgate A, Celenza A, et al. Anaphylaxis: clinical patterns, mediator release, and severity. J Allergy Clin Immunol. 2013;132(5):1141-9 e5.

3. Brown SG. Clinical features and severity grading of anaphylaxis. J Allergy Clin Immunol. 2004;114(2):371-6.

4. Victoria SC. Victorian Children’s Tool for Observation and Response (ViCTOR). Royal Childrens Hospital Clinical Practice Guidelines. 2023.

5. McLendon K SB. Anaphylaxis. StatPearls 2024 Jan.

6. Cardona V, Ansotegui IJ, Ebisawa M, El-Gamal Y, Fernandez Rivas M, Fineman S, et al. World allergy organization anaphylaxis guidance 2020. World Allergy Organ J. 2020;13(10):100472.

7. Dribin TE, Schnadower D, Spergel JM, Campbell RL, Shaker M, Neuman MI, et al. Severity grading system for acute allergic reactions: A multidisciplinary Delphi study. J Allergy Clin Immunol. 2021;148(1):173-81.

8. Chinthrajah RS, Jones SM, Kim EH, Sicherer SH, Shreffler W, Lanser BJ, et al. Updating the CoFAR Grading Scale for Systemic Allergic Reactions in Food Allergy. J Allergy Clin Immunol. 2022;149(6):2166-70 e1.

9. Ring J M, K. Incidence and Severity of anyphylactoid reactions to colloid volume substitute. Lancet. 1977:466.

10. Patel N, Chong KW, Yip AYG, Ierodiakonou D, Bartra J, Boyle RJ, et al. Use of multiple epinephrine doses in anaphylaxis: A systematic review and meta-analysis. The Journal of Allergy and Clinical Immunology. 2021;148(5):1307-15.

11. Miguel A Tejedor-Alonso SP-C. Worldwide incidence of fatal anaphylaxis: a systematic review and meta-analysis of observational studies.

12. Pumphrey R. Lessons for Management of anaphylaxis from a study of fatal reactions. Clincal and Experimental Immunology. 2000;30.

13. Costanzo G, Costanzo GAML, Del Moro L, Nappi E, Pelaia C, Puggioni F, et al. Mast Cells in Upper and Lower Airway Diseases: Sentinels in the Front Line. International Journal of Molecular Sciences. 2023;24(11):9771.

14. Kounis NG, Soufras GD, Hahalis G. Anaphylactic cardiac collapse, sudden death and the Kounis syndrome. J Postgrad Med. 2014;60(3):227-9.

15. Harper NJN, Cook TM, Garcez T, Lucas DN, Thomas M, Kemp H, et al. Anaesthesia, surgery, and life-threatening allergic reactions: management and outcomes in the 6th National Audit Project (NAP6). Br J Anaesth. 2018;121(1):172-88.

16. Mullins RJ, Wainstein BK, Barnes EH, Liew WK, Campbell DE. Increases in anaphylaxis fatalities in Australia from 1997 to 2013. Clin Exp Allergy. 2016;46(8):1099-110.

17. Euser AM, Zoccali C, Jager KJ, Dekker FW. Cohort Studies: Prospective versus Retrospective. Nephron Clinical Practice. 2009;113(3):c214-c7.

18. von Elm E, Altman DG, Egger M, Pocock SJ, Gøtzsche PC, Vandenbroucke JP. The Strengthening the Reporting of Observational Studies in Epidemiology (STROBE) statement: guidelines for reporting observational studies. The Lancet. 2007;370(9596):1453-7.

19. Turner PJ, Ansotegui IJ, Campbell DE, Cardona V, Carr S, Custovic A, et al. Updated grading system for systemic allergic reactions: Joint Statement of the World Allergy Organization Anaphylaxis Committee and Allergen Immunotherapy Committee. World Allergy Organization Journal. 2024;17(3).

20. Niggemann B, Beyer K. Time for a new grading system for allergic reactions? Allergy. 2016;71(2):135-6.

21. Kolawole H, Marshall SD, Crilly H, Kerridge R, Roessler P. Australian and New Zealand Anaesthetic Allergy Group/Australian and New Zealand College of Anaesthetists Perioperative Anaphylaxis Management Guidelines. Anaesth Intensive Care. 2017;45(2):151-8.

22. Ewan PW, Clark AT. Efficacy of a management plan based on severity assessment in longitudinal and case-controlled studies of 747 children with nut allergy: proposal for good practice. Clin Exp Allergy. 2005;35(6):751-6.

23. Hourihane JO, Grimshaw KE, Lewis SA, Briggs RA, Trewin JB, King RM, et al. Does severity of low-dose, double-blind, placebo-controlled food challenges reflect severity of allergic reactions to peanut in the community? Clin Exp Allergy. 2005;35(9):1227-33.

24. Sampson HA. Anaphylaxis and Emergency Treatment. Paediatrics 2003;111:1601-8.

25. Mueller H. Further experiences with severe allergic reactions to insect stings. N Engl J Med. 1959;261(8).

26. Anonymous. Croup (Laryngotracheobronchitis) Clinical Practice Guideline2024 June 2024. Available from: <https://www.rch.org.au/clinicalguide/guideline_index/croup_laryngotracheobronchitis/>.

27. Anonymous. Acute Asthma Clinical Practice GuidelineJune 2024. Available from: <https://www.rch.org.au/clinicalguide/guideline_index/asthma_acute/>.

28. Pederson KR, Peter. Perioperative Management Guideline2022.

29. Garvey LH, Mertes PM. Perioperative anaphylaxis-management and outcomes in NAP6. Br J Anaesth. 2018;121(1):120-3.

30. Stafford A, Turner PJ. Grading the severity of anaphylaxis. Curr Opin Allergy Clin Immunol. 2023;23(3):218-25.

31. Pumphrey RS. Fatal posture in anaphylactic shock. J Allergy Clin Immunol. 2003;112(2):451-2.

Organ failure in fatal anaphylaxis: a 20-year Australian population cohort study

**Statistical Analysis Plan (SAP)**

V1.0

20^th^ May 2025

UniMelb HREC 2023-27190-43989-3

National Coronial Information System Reference M0523

**Authors:**

**Dr Chris Selman**

Department of Critical Care

The University of Melbourne

Parkville, Victoria 3052 Australia

**Dr Ben McKenzie**

Department of Medicine

The University of Melbourne

Parkville, Victoria 3051 Australia

**1.0 Study Synopsis**

## Primary Objectives

To examine differences in primary organ failure that develop in patients who have fatal anaphylaxis after being exposed to different allergen trigger categories. We define patient allergen exposure to one of four categories of allergens: drug, insect, food, and radiocontrast, where ‘drug’ will be the reference group.

## Secondary Objectives

To compare the following outcomes by patient allergen exposure:

1. Time to symptoms from allergen exposure.
2. Time from symptom onset to severe symptoms.
3. Time from severe symptoms to cardiorespiratory arrest.
4. Time from arrest to death.
5. Whether the patient required tracheal intubation.
   1. If required tracheal intubation, the timing of tracheal intubation.
   2. Time to tracheal intubation from cardiorespiratory arrest.
6. Whether the patient experienced vomiting or aspiration.
7. Whether the patient received cardioversion (defined as no shocks vs at least one shock).
8. Change in posture.
9. Frequency of deaths per year over time (by allergen exposure and by age group).

To compare primary organ failure by the following complications:

1. Difficult airway (FONA, failed intubation, or 3 or more attempts at intubation)
2. FONA (front of neck access)
3. Vomiting or aspiration
4. Pneumothorax (dichotomised to none OR one+/- both sides)
5. Cardioversion

## Study Population

This is a retrospective study of consecutive anaphylaxis deaths of all ages in Australia between 1^st^ January 2003 and 31^st^ December 2022.

# General Statistical Methodology

## Analysis Software

All analyses will be performed using Stata Release 19.0 or later.

## Data verification

A thorough data cleaning and data checking process of the database that will enable detection and correction of discrepancies will be conducted prior to analysis.

## Adjustment for Multiplicity

No formal adjustments for multiplicity of testing will be applied, with results interpreted based on the magnitude of the estimates and the 95% confidence intervals (CIs) rather than focussing on p-values.

## Handling of Missing Data

The analyses for all outcomes will be conducted using participants with available data.

However, if there are >10% missing data in the primary or key secondary outcomes (or exposure variables), we will conduct a sensitivity analysis under a plausible assumption regarding the missingness mechanism. Multiple imputation will be used to handle the missing data in all models used for outcomes. Imputations will be generated using chained equations, also known as fully conditional specification, with 50 imputed datasets and 10 iterations between each imputation. Any variables that are predictors of missingness and/or associated with the incomplete outcomes will be included as auxiliary variables in the imputation model. Estimates of interest will be obtained using Rubin’s rules, and will be reported with 95% confidence intervals and *p*-values.

For the analysis of time-to-event outcomes, rather than using multiple imputation, we will handle missing data (i.e. those who did not experience the outcome) by censoring those patients at the time of death.

# Descriptive Statistics

## Baseline Characteristics

The following variables will be summarised at baseline. Categorical variables will be summarised as the number and percentage, and continuous variables as mean, standard deviation (SD) and range.

**Baseline variables:**

- Sex
- Age (continuous)
- Age (categorical): 0-4, 5-17, 18-39, 40-59 and >60 years
- Pre-existing asthma/COPD status
- Pre-existing anti-hypertensive documented
- ASA score
- Any known allergies
- Known allergy to trigger
- Location of allergen exposure

# Analysis of the Primary Outcome

## Primary Analysis

*Causal effect of allergen exposure trigger on primary organ failure*

The analysis will include allergen trigger as the exposure variable of interest and primary organ failure (defined as whether the primary organ failure was upper airway, bronchospasm, or cardiovascular) as the outcome in three models (where each type of organ failure is a separate outcome). We will fit a logistic regression model, adjusted for the set of confounders listed below, and estimate an adjusted odds ratio reported with its 95% confidence interval (CI) and p-value. If we identify effect modifiers, an adjusted marginal odds ratio will be reported, estimated following model fitting using g-computation. We will also report the number and percentage meeting the outcome in each exposure group. Since the exposure variable has multiple categories, we will present each pairwise odds ratio.

**Confounding variable set:**

- Age

**Potential effect modifiers:**

- Age
- Asthma/COPD status
- Antihypertensive Use
- ASA Score

The directed acyclic graph below describes the assumed causal relationship between allergen trigger and primary organ failure.

**Set of confounders**

**Allergen trigger**

**Primary organ failure**

## Sensitivity Analyses

A sensitivity analyses will be conducted that uses multiple imputation to handle missing data if there is >10% missing data in the analysis model. The results will be compared to a complete case analysis.

We will also present the unadjusted estimate (i.e. unadjusted odds ratio) for the analysis of the primary outcome.

## Subgroup Analyses

As an exploratory analysis, we will also estimate the effect of allergen trigger on primary organ failure in the following subgroups:

- Age <18 vs 18+ years
- Age <40 (including children) vs 40 or above

# Secondary Outcomes

## 6.1 Main Analysis

All secondary objectives will be analysed as descriptive objectives and will not adjust for any confounders.

One objective is to compare the following outcomes between patient allergen exposure including:

1. Time to symptoms from allergen exposure (***ordinal outcome***).
2. Time to symptom onset to severe symptoms (***continuous outcome***).
3. Time to severe symptoms to cardiorespiratory arrest (***continuous outcome***).
4. Time to arrest to death (***ordinal outcome***).
5. Whether the patient required tracheal intubation (***binary outcome***).
   1. If required tracheal intubation, the timing of tracheal intubation (***nominal outcome***).
   2. Time to tracheal intubation from cardiorespiratory arrest among those not already intubated in theatre (***continuous outcome***).
6. Whether the patient experienced vomiting or aspiration (***binary outcome***).
7. Whether the patient received cardioversion (***binary outcome***).
8. Change in posture (***nominal outcome***).
9. Frequency of deaths per year over time by (i) allergen exposure and by (ii) age group (<18, 18-40, 40-50, 50-60, >60 (***count outcome***).

Another objective is to compare primary organ failure by the following complications:

1. Difficult airway
2. FONA (front of neck access)
3. Vomiting or aspiration
4. Pneumothorax (dichotomised to none OR one+/- both sides)
5. Cardioversion

Binary outcomes will be analysed using a similar approach to that described for the primary outcome. The unadjusted odds ratio will be estimated using a logistic regression model, reported with its 95% CI and p-value. We will also report the number and percentage meeting the outcome in each exposure group.

Continuous outcomes will be summarised by exposure group using mean and standard deviation. The unadjusted mean difference will be estimated using a linear regression model with robust standard errors, reported with its 95% CI and p-value. If the outcome appears skewed, we will instead estimate a median difference using quantile regression.

Count outcomes will be summarised by exposure group for each time point (i.e. reporting the number of deaths in each year by exposure). The adjusted incidence rate ratio will be estimated using Poisson regression, reported with the 95% CI and p-value. Given that we suspect that the rate of death is not constant over time, an exposure x time interaction term will be included in the model. If there is evidence of non-constant rate over time, we will present the marginal adjusted incidence rate ratio. If there is any evidence of overdispersion, we will instead use negative binomial regression to estimate the incidence rate ratios.

Nominal outcomes will be compared between exposure group by using cross-tabulations. If there are >5 expected counts in each cell, we will use the Chi-Square test as a measure of association.

**6.2 Sensitivity Analyses**

A sensitivity analyses will be conducted that uses multiple imputation to handle missing data if there is >10% missing data in the analysis model.

# 7.References

[1] Lee KJ, Carlin JB, Simpson JA, Moreno-Betancur M. Assumptions and analysis planning in studies with missing data in multiple variables: moving beyond the MCAR/MAR/MNAR classification. *Int J Epidemiol*. 2023;52(4):1268-1275. doi:10.1093/ije/dyad008

**Primary Outcome Complete Analysis**

Primary outcome complete analysis vs multiple imputation analysis is published for statistical completeness and transparency but shows little difference in the significance or strength of results.

**Table S2a**: Summary of overall average causal effect of allergen exposure on primary organ failure (bronchospasm)

| **Exposure or Contrast** | **Number (%) with bronchospasm as primary organ failure** | **Complete case analysis** | | **Multiple imputation** | |
| --- | --- | --- | --- | --- | --- |
|  |  | **Odds ratio (95% CI)** | **p-value** | **Odds ratio (95% CI)** | **p-value** |
| **Drug** | 68/115 (59.1%) |  |  |  |  |
| **Insect** | 9/37 (24.3%) |  |  |  |  |
| **Food** | 55/58 (94.8%) |  |  |  |  |
| **Contrast** | 13/27 (48.1%) |  |  |  |  |
| **Adjusted estimates (causal effects)** | | | | | |
| **Insect v Drug** | - | 0.178 (0.071 to 0.448) | <0.001 | 0.185 (0.073 to 0.466) | <0.001 |
| **Food v Drug** | - | 1.980 (0.653 to 6.008) | 0.226 | 2.867 (0.641 to 12.827) | 0.167 |
| **Contrast v Drug** | - | 0.339 (0.130 to 0.887) | 0.028 | 0.445 (0.168 to 1.175) | 0.101 |
| **Food v Insect** | - | 11.137 (3.038 to 40.826) | <0.001 | 15.530 (3.166 to 76.186) | 0.001 |
| **Contrast v Insect** | - | 1.909 (0.536 to 6.803) | 0.317 | 2.410 (0.648 to 8.964) | 0.187 |
| **Contrast v Food** | - | 0.171 (0.044 to 0.671) | 0.012 | 0.155 (0.027 to 0.901) | 0.038 |
| **Unadjusted estimates (associations)** | | | | | |
| **Insect v Drug** | - | 0.222 (0.096 to 0.514) | <0.001 | 0.222 (0.094 to 0.528) | 0.001 |
| **Food v Drug** | - | 12.672 (3.741 to 42.924) | <0.001 | 12.399 (3.615 to 42.528) | <0.001 |
| **Contrast v Drug** | - | 0.642 (0.277 to 1.489) | 0.302 | 0.585 (0.253 to 1.353) | 0.209 |
| **Food v Insect** | - | 57.037 (14.298 to 227.535) | <0.001 | 55.731 (13.904 to 223.378) | <0.001 |
| **Contrast v Insect** | - | 2.889 (0.996 to 8.379) | 0.051 | 2.631 (0.905 to 7.644) | 0.075 |
| **Contrast v Food** | - | 0.051 (0.013 to 0.202) | <0.001 | 0.047 (0.012 to 0.185) | <0.001 |

**Table S2b**: Summary of overall average causal effect of allergen exposure on primary organ failure (cardiovascular)

| **Exposure or Contrast** | **Number (%) with cardiovascular as primary organ failure** | **Complete case analysis** | | **Multiple imputation** | |
| --- | --- | --- | --- | --- | --- |
|  |  | **Odds ratio (95% CI)** | **p-value** | **Odds ratio (95% CI)** | **p-value** |
| **Drug** | 40/115 (34.8%) |  |  |  |  |
| **Insect** | 21/37 (56.8%) |  |  |  |  |
| **Food** | 0/58 (0.0%) |  |  |  |  |
| **Contrast** | 12/27 (44.4%) |  |  |  |  |
| **Adjusted estimates (causal effects)** | | | | | |
| **Insect v Drug** | - | 2.920 (1.208 to 7.055) | 0.018 | 3.076 (1.296 to 7.305) | 0.011 |
| **Food v Drug** | - | Cannot be estimated | N/A | Cannot be estimated | N/A |
| **Contrast v Drug** | - | 1.589 (0.668 to 3.781) | 0.293 | 1.503 (0.612 to 3.691) | 0.371 |
| **Food v Insect** | - | Cannot be estimated | N/A | Cannot be estimated | N/A |
| **Contrast v Insect** | - | 0.544 (0.178 to 1.667) | 0.285 | 0.489 (0.157 to 1.516) | 0.213 |
| **Contrast v Food** | - | Cannot be estimated | N/A | Cannot be estimated | N/A |
| **Unadjusted estimates (associations)** | | | | | |
| **Insect v Drug** | - | 2.461 (1.156 to 5.237) | 0.019 | 2.591 (1.238 to 5.422) | 0.012 |
| **Food v Drug** | - | Cannot be estimated | N/A | Cannot be estimated | N/A |
| **Contrast v Drug** | - | 1.500 (0.641 to 3.511) | 0.350 | 1.535 (0.646 to 3.648) | 0.331 |
| **Food v Insect** | - | Cannot be estimated | N/A | Cannot be estimated | N/A |
| **Contrast v Insect** | - | 0.610 (0.224 to 1.656) | 0.332 | 0.592 (0.212 to 1.655) | 0.316 |
| **Contrast v Food** | - | Cannot be estimated | N/A | Cannot be estimated | N/A |

**Table S2c**: Summary of overall average causal effect of allergen exposure on primary organ failure (Upper airway)

| **Exposure or Contrast** | **Number (%) with upper airway as primary organ failure** | **Complete case analysis** | | **Multiple imputation** | |
| --- | --- | --- | --- | --- | --- |
|  |  | **Odds ratio (95% CI)** | **p-value** | **Odds ratio (95% CI)** | **p-value** |
| **Drug** | 7/115 (6.1%) |  |  |  |  |
| **Insect** | 7/37 (18.9%) |  |  |  |  |
| **Food** | NR |  |  |  |  |
| **Contrast** | NR |  |  |  |  |
| **Adjusted estimates (causal effects)** | | | | | |
| **Insect v Drug** | - | 3.534 (0.927 to 13.468) | 0.064 | 3.610 (1.186 to 10.991) | 0.024 |
| **Food v Drug** | - | 2.946 (0.393 to 22.082) | 0.292 | 2.113 (0.277 to 16.150) | 0.468 |
| **Contrast v Drug** | - | 6.935 (2.183 to 22.032) | 0.001 | 2.565 (0.566 to 11.621) | 0.220 |
| **Food v Insect** | - | 0.834 (0.127 to 5.486) | 0.849 | 0.585 (0.079 to 4.343) | 0.598 |
| **Contrast v Insect** | - | 1.962 (0.560 to 6.881) | 0.291 | 0.711 (0.131 to 3.844) | 0.690 |
| **Contrast v Food** | - | 2.354 (0.329 to 16.828) | 0.392 | 1.214 (0.098 to 15.087) | 0.879 |
| **Unadjusted estimates (associations)** | | | | | |
| **Insect v Drug** | - | 3.600 (1.171 to 11.067) | 0.025 | 3.083 (1.129 to 8.416) | 0.028 |
| **Food v Drug** | - | 0.842 (0.209 to 3.382) | 0.808 | 0.632 (0.156 to 2.566) | 0.520 |
| **Contrast v Drug** | - | 1.234 (0.242 to 6.303) | 0.800 | 1.494 (0.317 to 7.041) | 0.610 |
| **Food v Insect** | - | 0.234 (0.056 to 0.971) | 0.045 | 0.205 (0.050 to 0.847) | 0.029 |
| **Contrast v Insect** | - | 0.343 (0.065 to 1.801) | 0.206 | 0.485 (0.098 to 2.387) | 0.371 |
| **Contrast v Food** | - | 1.467 (0.230 to 9.333) | 0.685 | 2.363 (0.379 to 14.722) | 0.356 |

**Association of complications with primary outcome and allergen trigger category**

As a condition of ethics approval, death counts of less than five cannot be directly reported. Where this has occurred in tabular reporting, we have abbreviated this to Not Reportable (NR).

**Table S3:** Association between complication and primary organ failure

|  | **Number (%) with specified primary organ failure** | **Number (%) without specified primary organ failure** | **Complete case analysis** | | **Multiple imputation** | |
| --- | --- | --- | --- | --- | --- | --- |
|  |  |  | **Odds ratio (95% CI)** | **p-value** | **Odds ratio (95% CI)** | **p-value** |
| **Primary Organ Failure: Bronchospasm** | | | | | | |
| **Difficult airway** | 21/29 (72.4%) | 101/160 (63.1%) | 1.533 (0.639 to 3.680) | 0.338 | 1.523 (0.668 to 3.473) | 0.316 |
| **Cricothyroidotomy** | 5/13 (38.5%) | 122/193 (63.2%) | 0.364 (0.115 to 1.155) | 0.086 | 0.380 (0.130 to 1.117) | 0.078 |
| **Vomiting or aspiration** | 37/53 (69.8%) | 116/197 (58.9%) | 1.615 (0.842 to 3.098) | 0.149 | 1.774 (0.944 to 3.334) | 0.075 |
| **Pneumothorax** | 11/18 (61.1%) | 129/209 (61.7%) | 0.975 (0.363 to 2.617) | 0.959 | 1.014 (0.363 to 2.835) | 0.979 |
| **Cardioversion** | 26/41 (63.4%) | 32/46 (69.6%) | 0.758 (0.310 to 1.853) | 0.544 | N/A | N/A |
| **Primary Organ Failure: Cardiovascular** | | | | | | |
| **Difficult airway** | NR | 53/160 (33.1%) | 0.150 (0.034 to 0.653) | 0.011 | 0.206 (0.053 to 0.807) | 0.024 |
| **Cricothyroidotomy** | NR | 55/193 (28.5%) | 0.753 (0.200 to 2.839) | 0.675 | 0.974 (0.273 to 3.475) | 0.967 |
| **Vomiting or aspiration** | 12/53 (22.6%) | 61/197 (31.0%) | 0.653 (0.321 to 1.328) | 0.239 | 0.618 (0.313 to 1.220) | 0.165 |
| **Pneumothorax** | 5/18 (27.8%) | 60/209 (28.7%) | 0.955 (0.326 to 2.796) | 0.933 | 0.992 (0.344 to 2.858) | 0.988 |
| **Cardioversion** | 12/41 (29.3%) | 12/46 (26.1%) | 1.172 (0.457 to 3.005) | 0.740 | N/A | N/A |
| **Primary Organ Failure: Upper airway** | | | | | | |
| **Difficult airway** | 6/29 (20.7%) | 6/160 (3.8%) | 6.696 (1.990 to 22.531) | 0.002 | 3.401 (1.126 to 10.269) | 0.030 |
| **Cricothyroidotomy** | 5/13 (38.5%) | 16/193 (8.3%) | 6.914 (2.023 to 23.629) | 0.002 | 5.530 (1.627 to 18.804) | 0.006 |
| **Vomiting or aspiration** | NR | 20/197 (10.2%) | 0.722 (0.236 to 2.212) | 0.569 | 0.653 (0.216 to 1.971) | 0.449 |
| **Pneumothorax** | NR | 20/209 (9.6%) | 1.181 (0.253 to 5.513) | 0.832 | 1.133 (0.206 to 6.232) | 0.886 |
| **Cardioversion** | NR | NR | 1.737 (0.276 to 10.948) | 0.557 | N/A | N/A |

**Table S4:** Association between complication and allergen exposure

|  | **Number (%) with complication among those with allergen exposure** | **Number (%) with complication among those without allergen exposure** | **Complete case analysis** | | **Multiple imputation** | |
| --- | --- | --- | --- | --- | --- | --- |
|  |  |  | **Odds ratio (95% CI)** | **p-value** | **Odds ratio (95% CI)** | **p-value** |
| **Allergen Exposure: Drug** | | | | | | |
| **Difficult airway** | 14/134 (10.4%) | 13/103 (12.6%) | 0.808 (0.362 to 1.803) | 0.602 | 0.931 (0.421 to 2.058) | 0.860 |
| **Cricothyroidotomy** | 5/141 (3.5%) | 7/112 (6.2%) | 0.551 (0.170 to 1.787) | 0.321 | 0.630 (0.211 to 1.880) | 0.406 |
| **Vomiting or aspiration** | 19/179 (10.6%) | 39/172 (22.7%) | 0.405 (0.223 to 0.734) | 0.003 | 0.411 (0.228 to 0.740) | 0.003 |
| **Pneumothorax** | 17/161 (10.6%) | 10/162 (6.2%) | 1.794 (0.795 to 4.049) | 0.159 | 1.857 (0.826 to 4.175) | 0.134 |
| **Cardioversion** | 34/56 (60.7%) | 17/55 (30.9%) | 3.455 (1.577 to 7.566) | 0.002 | N/A | N/A |
| **Allergen Exposure: Food** | | | | | | |
| **Difficult airway** | 6/51 (11.8%) | 21/186 (11.3%) | 1.048 (0.399 to 2.751) | 0.925 | 0.853 (0.334 to 2.177) | 0.739 |
| **Cricothyroidotomy** | NR | 10/203 (4.9%) | 0.804 (0.171 to 3.792) | 0.783 | 0.561 (0.114 to 2.764) | 0.477 |
| **Vomiting or aspiration** | 26/64 (40.6%) | 32/287 (11.1%) | 5.452 (2.934 to 10.132) | 0.000 | 5.295 (2.876 to 9.749) | 0.000 |
| **Pneumothorax** | NR | 23/264 (8.7%) | 0.762 (0.253 to 2.293) | 0.629 | 0.698 (0.235 to 2.069) | 0.516 |
| **Cardioversion** | 6/21 (28.6%) | 45/90 (50.0%) | 0.400 (0.142 to 1.124) | 0.082 | N/A | N/A |
| **Allergen Exposure: Insect** | | | | | | |
| **Difficult airway** | 5/24 (20.8%) | 22/213 (10.3%) | 2.285 (0.776 to 6.724) | 0.134 | 1.679 (0.571 to 4.938) | 0.344 |
| **Cricothyroidotomy** | 5/28 (17.9%) | 7/225 (3.1%) | 6.770 (1.988 to 23.057) | 0.002 | 5.302 (1.512 to 18.586) | 0.009 |
| **Vomiting or aspiration** | 7/63 (11.1%) | 51/288 (17.7%) | 0.581 (0.250 to 1.348) | 0.206 | 0.546 (0.236 to 1.264) | 0.158 |
| **Pneumothorax** | NR | 23/263 (8.7%) | 0.745 (0.248 to 2.241) | 0.601 | 0.756 (0.253 to 2.263) | 0.617 |
| **Cardioversion** | 3/20 (15.0%) | 48/91 (52.7%) | 0.158 (0.043 to 0.577) | 0.005 | N/A | N/A |
| **Allergen Exposure: Contrast** | | | | | | |
| **Difficult airway** | NR | 25/209 (12.0%) | 0.566 (0.127 to 2.531) | 0.457 | 0.676 (0.165 to 2.776) | 0.586 |
| **Cricothyroidotomy** | 0/34 (0.0%) | 12/219 (5.5%) | Cannot be estimated | N/A | N/A | N/A |
| **Vomiting or aspiration** | 6/45 (13.3%) | 52/306 (17.0%) | 0.751 (0.303 to 1.867) | 0.538 | 0.747 (0.299 to 1.866) | 0.532 |
| **Pneumothorax** | NR | 25/280 (8.9%) | 0.498 (0.114 to 2.180) | 0.354 | 0.508 (0.114 to 2.261) | 0.374 |
| **Cardioversion** | 8/14 (57.1%) | 43/97 (44.3%) | 1.674 (0.540 to 5.193) | 0.372 | N/A | N/A |
